# Rab27a GTPase and its effector Myosin Va are host factors required for efficient Oropouche virus cell egress

**DOI:** 10.1371/journal.ppat.1012504

**Published:** 2024-08-30

**Authors:** Juan O. Concha, Kristel Gutierrez, Natalia Barbosa, Roger L. Rodrigues, Andreia N. de Carvalho, Lucas A. Tavares, Jared S. Rudd, Cristina S. Costa, Barbara Y. G. Andrade, Enilza M. Espreafico, Colin M. Crump, Luis L. P. daSilva

**Affiliations:** 1 Virus Research Center, Ribeirão Preto Medical School, University of São Paulo, Ribeirão Preto, São Paulo, Brazil; 2 Department of Cell and Molecular Biology, Ribeirão Preto Medical School, University of São Paulo, Ribeirão Preto, São Paulo, Brazil; 3 Department of Pathology, University of Cambridge, Cambridge, United Kingdom; University of Pittsburgh, UNITED STATES OF AMERICA

## Abstract

Oropouche fever, a debilitating illness common in South America, is caused by Oropouche virus (OROV), an arbovirus. OROV belongs to the *Peribunyaviridae* family, a large group of RNA viruses. Little is known about the biology of *Peribunyaviridae* in host cells, especially assembly and egress processes. Our research reveals that the small GTPase Rab27a mediates intracellular transport of OROV induced compartments and viral release from infected cells. We show that Rab27a interacts with OROV glycoproteins and colocalizes with OROV during late phases of the infection cycle. Moreover, Rab27a activity is required for OROV trafficking to the cell periphery and efficient release of infectious particles. Consistently, depleting Rab27a’s downstream effector, Myosin Va, or inhibiting actin polymerization also hinders OROV compartments targeting to the cell periphery and infectious viral particle egress. These data indicate that OROV hijacks Rab27a activity for intracellular transport and cell externalization. Understanding these crucial mechanisms of OROV’s replication cycle may offer potential targets for therapeutic interventions and aid in controlling the spread of Oropouche fever.

## Author summary

Since its identification in the early 1960s, Oropouche virus (OROV) has caused over 30 epidemics in Central and South America, infecting an estimated half a million people. Environmental changes, urbanization, and increased travel increase the risk of OROV spreading beyond its endemic regions. The lack of vaccines and specific antiviral treatments highlights the urgent need to better understand OROV biology. Previous research shows that OROV assembles in the Golgi complex of human cells, forming vesicular compartments that transport viral particles to the cell periphery, leading to virus spread. Our findings reveal that OROV hijacks the Rab27a GTPase and its effector, the motor protein Myosin Va, to facilitate viral egress. Specifically, we show that Rab27a associates with OROV glycoproteins and, along with Myosin Va, is found in viral-induced compartments at late infection stages. Depleting Rab27a or Myosin Va, or expressing defective Rab27a mutants, compromises the release of infectious viral particles. We propose that vesicles containing OROV exploit Rab27a/Myosin Va-mediated transport to competently reach the cell periphery and fuse with the plasma membrane, enabling their release and viral transmission to other cells. Understanding the molecular mechanisms underlying OROV replication will be crucial for developing future anti-OROV drugs, which are currently unavailable.

## Introduction

The *Bunyavirales* is an order of enveloped, negative-sense single-stranded RNA viruses that contain a segmented genome of 2 to 6 segments [1,2]. This large viral order comprises several virus families, including the *Peribunyaviridae* with eight genera, one of which is the *Orthobunyavirus*, with at least 103 species, such as Oropouche virus (OROV). OROV is an arbovirus that is the etiologic agent of Oropouche fever, an acute febrile zoonotic disease with clinical symptoms common to other arboviruses, which in some cases may present manifestations of neurological disorders such as meningitis and meningoencephalitis [3–5], and that affect several countries in South America, especially in the Amazon region [4,6].

Similar to other members of the *Peribunyaviridae* family, the OROV genome is composed of a small (S), a medium (M), and a large (L) segment. The S segment encodes the nucleocapsid (N) protein which wraps the RNA. The M segment encodes the glycoprotein precursor, which is cleaved into the glycoproteins Gn and Gc. Finally, the L segment encodes the RNA-dependent RNA polymerase (RdRp) or L protein. In addition to these four structural proteins, as for most members of this family, OROV also encodes two non-structural proteins: one from the S segment (NSs) and one from the M segment (NSm) [7–9].

Concerning the cell biology of OROV, it was shown that this virus enters cells via clathrin-coated vesicles [10], and the low-density lipoprotein receptor-related protein (Lrp1) has been shown to act as an entry receptor [11]. OROV endocytic entry depends on endosomal acidification to activate the membrane fusion process [10]. After the fusion of the virus envelope with the endosomal membrane, it is believed that the viral genome is released in the cytosol, and the expression of viral proteins and replication of the genome takes place. The glycoprotein precursor is co-translationally inserted in the endoplasmic reticulum giving rise to the glycoproteins [12] that are directed towards Golgi-derived compartments, where viral assembly takes place [13]. However, the process of transport and egress of viral particles remains mostly unknown.

OROV budding is thought to require the host ESCRT machinery [13], and ESCRT subunits, such as CHMP6, are recruited to the Golgi area upon OROV glycoprotein expression [12]. The ESCRT machinery participates in the formation of intraluminal vesicles (ILVs) during the biogenesis of multivesicular bodies (MVBs), which are released from cells as extracellular vesicles (EVs) when MVBs fuse to the plasma membrane [14]. Accordingly, it would be plausible to think that OROV externalization may involve host factors required for the extracellular release of EVs. Therefore, this study aimed to investigate whether OROV externalization requires Rab27 GTPases, which are known to regulate secretion mediated by MVBs [15–17], melanosomes [18,19], other endo-lysosomal related organelles [20–22], and the release of certain viruses like HSV-1 and HEV [23–26].

Here, we demonstrate that Rab27a and Rab27b GTPases are important for efficient OROV production in human cells. Specifically, we show that Rab27a interacts with OROV glycoproteins, colocalizes to OROV assembly compartments, and is important for their effective transport to the cell surface. Finally, we show that Rab27a protein is released from cells in association with the viral particles, and that the Rab27a downstream effectors Myosin Va protein (MyoVa) together with actin filaments are important for OROV intracellular transport and release. We conclude that OROV uses the Rab27a-MyoVa-Actin system for effective egress from infected cells.

## Materials and methods

### Oropouche virus stocks

The OROV stock used in this study belongs to the BeAn19991 reference strain and was kindly provided by Dr. Luiz Tadeu Moraes Figueiredo (CPV FMRP-USP). The virus was propagated in Vero cell culture through four serial passages. Stocks were made from the supernatant of these cultures, which were filtered through 0.22 μm pores (Millipore, CO, USA), aliquoted, and frozen in a freezer at -80°C. An aliquot was thawed and titrated using the plaque forming units (PFU) assay method.

### Cell culture, transfections, and RNA interference (RNAi)

HeLa, HEK293T and Vero CCL81 cells were acquired from ATCC (American Type Culture Collection). The FO and RO human fibroblasts were previously described [27]. Briefly, human skin derived fibroblasts were isolated from a Griscelli Syndrome type I/Elejalde syndrome patient carrying a non-sense mutation in the MYO5A gene (termed FO cells). The mutation renders these cells null for Myosin Va protein. Normal human fibroblasts, age-paired with FO, were isolated under the same conditions and used as control (termed RO cells). Cells were cultured in DMEM-Dulbecco’s Modified Eagle Medium (Thermo Fisher Scientific) supplemented with 10% fetal bovine serum, 100 U/mL penicillin, and 100 μg/mL streptomycin, at 37°C, 5% (v/v) CO_2_.

Plasmid DNA transfections were performed with the lipofectamine 2000 reagent, following the manufacturer’s instructions. The specific siRNAs for Rab27 proteins were commercially purchased (Sigma Aldrich), as follows: Rab27a 5’-GAUCUUCUCUAUGAUUGAU[dT][dT]; Rab27b 5’-CAGUCAACAGAGCUUCUUA[dT][dT]. siRNA transfections were performed with oligofectamine (Life Technologies, Carlsbad, CA, USA), following the manufacturer’s instructions.

### Infection with OROV for PFU assays and western blot analysis

To determine the replication curve of OROV in HeLa cells, semi-confluent monolayers of cells were washed with ice-cold DPBS buffer and inoculated with the OROV stock. The cells were incubated with the viral inoculum for 2 hours at 4°C under gentle agitation, for adsorption of viral particles to the plasma membrane. After this time, the inoculum was removed and the cell monolayers were washed five times with ice-cold DPBS and then warm DMEM supplemented with 2% FBS was added to promote synchronized viral entry. Subsequently, cells were incubated at 37°C in a humidified incubator containing 5% CO_2_. After the stated infection times, the supernatant was collected and clarified by centrifugation at 4°C. The clarified supernatants were transferred to a fresh tube and either immediately used to concentrate viral particles by ultracentrifugation (see below), or stored at -80°C for later titration. Monolayers of infected cells were washed with DPBS to remove the culture media and then incubated for 5 minutes in DPBS supplemented with 2 mM EDTA to detach the cells. The detached cells were transferred to a tube on ice and spun at 4°C to lower the cells. The DPBS was removed from the cell pellets and these were stored at -80°C for later preparation of cell lysates for western blot analysis. For cell lysis, the cell pellets were resuspended on ice with 50 uL of lysis buffer (50 mM Tris-HCl, pH 7.5, 150 mM NaCl, glycerol 10% (v/v), 5 mM EDTA, 1% (v/v) Triton X-100 supplemented with protease inhibitor) and incubated for 20 min on ice. Then, the cell lysate was centrifuged at 16,000 x g for 20 min at 4°C, the supernatant was transferred to another tube, and sample buffer 5x supplemented with beta-mercaptoethanol was added. These samples were stored at -80°C or immediately heated to 95°C for 5 minutes for western blot analysis.

### Concentration of viral particles by ultracentrifugation

The collected and clarified supernatant samples were centrifuged at 12,000 × g for 10 minutes at 4°C to remove cellular debris, and then ultracentrifuged at 100,000 × g for 2 hours at 4°C. The resulting virus-containing pellets were resuspended in buffer, heated for 5 minutes at 95°C, and analyzed by immunoblotting.

### PFU assay

Titration of infectious virus was performed on Vero CCL81 cells at 80–90% confluence in a 24-well plate. The clarified supernatant samples stored at -80°C were thawed on ice for 45 min and serially diluted 5-fold in DMEM without fetal bovine serum (FBS). Cell monolayers were washed with DPBS (one time) and infected with 100 μl of the serial dilutions for 1 hour at 37°C under gentle shaking every 15 minutes. After, the infected cells were washed with DPBS and then overlaid with 1,5% carboxymethylcellulose (CMC) in DMEM medium supplemented with 2% FBS. 72 hours after infection, cells were fixed with 10% formaldehyde for 1 hour, washed with water and stained with crystal violet to visualize plaques, which were counted and virus yield was calculated and expressed as PFU/mL (Plaque Forming Units per mL).

### Immunofluorescence, Confocal Microscopy

Cells were plated onto coverslips in 24-well plates and infected with OROV (MOI = 4) as described above. At different h.p.i indicated in the Figures, cells were washed with PBS and fixed with 4% paraformaldehyde (EM Sciences) in PBS for 15 minutes at room temperature. Afterwards, the cells were washed with PBS and permeabilized with 0.01% saponin (Sigma-Aldrich) in a blocking solution (0.2% porcine gelatine in PBS) for 30 minutes at 37°C. After blocking, cells were incubated with primary and secondary antibodies for 30 minutes at 37°C in a blocking solution supplemented with 0.01% saponin. Between antibody incubations, cells were washed with PBS. Finally, the coverslips were mounted on glass slides (Knittel Glase) using Fluormount G (EM Sciences) and the material was observed and documented using a Zeiss LSM 780 confocal microscope available at the Multiuser Laboratory of Multiphoton Microscopy (LMMM) at Ribeirão Preto Medical School. Post-acquisition image processing was performed with ImageJ 1.53t [28]. Colocalization analyses by immunofluorescence were conducted using sets of images of the same cells (Z-stack, with 0.3-μm intervals) for each marker. Quantification was performed using ImageJ and the colocalization threshold plugin to determine the Manders’ coefficients (tM) for each channel. The Manders’ coefficients range from 0 to 1, corresponding to no overlap and complete overlap, respectively. Scores are calculated for pixels above an automatically determined threshold for both channels, according to an algorithm previously described [29].

### Antibodies

The mouse anti-OROV antiserum (kindly donated by Dr. Luis Tadeu Moraes Figueiredo) was used for immunofluorescence, western blot, and immunoelectron microscopy assays. This polyclonal antiserum detects OROV Gc and N proteins [13], but not Gn, as previously shown [12]. Affinity purified rabbit anti-MyoVa medial-tail domain previously described [30], rabbit anti-Rab27a (Cell Signaling, D7Z9Q), rabbit anti-Rab27b (Proteintech, 134 2-1-AP), rabbit anti-GAPDH (Sigma-Aldrich, G9545), sheep anti-TGN46 (AbD Serotec, AHP5000), mouse monoclonal anti-TfR (life technology, 136800), mouse monoclonal anti-actin (Santa cruz biotechnology, sc-47778), mouse anti-AP1γ1 (BD biosciences, 610386), and rabbit anti-CD9 (Abcam, 92726), rabbit mAb anti-HA tag (Cell Signaling, C29F4), mouse monoclonal anti-GFP (Santa Cruz, B-2, sc-9996). Secondary antibodies that were used in immunofluorescence assays are conjugated to Alexa Fluor 488, Alexa Fluor 594, or Alexa Fluor 647 and were purchased from Thermo Scientific (Rockford, IL). For western blot assays, secondary antibodies conjugated to peroxidase (GE Life sciences) were used.

### shRNA cell line generation

To produce lentivirus encoding shRNA, HEK-293T cells growing in a 10 cm dish, at 70% confluence, were transfected with either 3 μg of control shRNA-containing lentivirus plasmid (Mission TRC2 pLKO.5-pure non-mammalian shRNA control plasmid, catalog no. SHC202; Sigma-Aldrich), 3 μg of Rab27a shRNA-containing lentivirus plasmid (target sequence: 5’ GCTGCCAATGGGACAAACATA 3’) kindly donated by Dr Patricia V. Burgos, or 3 μg of lentivirus plasmid containing shRNA against MyoVa (target sequence: 5’ CGGATTTGAAACATTTGAGAT 3’), from Sigma-Aldrich (TRCN0000059490), along with 2.25 μg of psPAX2 (catalog #12259; Addgene) and 0.75 μg of pMD2.G (catalog #12259; Addgene), using 18 μl of 25 kDa linear PEI (1 mg/ml stock solution). After 15 h post-transfection, the culture medium was removed and 6 mL of DMEM medium supplemented with 2% FBS (without antibiotics) was added to the plate. Virus supernatants were subsequently collected for 2 days and cleared by centrifugation for 10 min at 2000 x g at 4°C. After centrifugation, the viral supernatant was added to the HeLa cell monolayer (70% confluence) growing in another 10 cm dish. Cells were then incubated for 120 h under puromycin selection followed by western blot analysis to check knockdown efficiency.

### Generation of stable HeLa cell lines expressing inducible wild type Rab27a and its GTP and GDP looked forms

To allow inducible expression of Rab27a and mutant forms in HeLa cells using a piggyBac transposon-based mammalian cell expression system [31], the human coding sequence for Rab27a (WT or mutants) were amplified by PCR from the original vectors: GFP-Rab27a(WT), GFP-Rab27a(Q78L) and GFP-Rab27a(T23N), kindly donated by Patricia Burgos (Universidad San Sebastian—Chile). The PCR fragments were then inserted into the NheI/HindIII sites of the PB-TSW vector, resulting in the PB-TRab27a(WT), PB-TRab27a(Q78L) and PB-TRab27a(T23N) constructs. The original PB-TSW, a generous gift from Dr Stephen Graham (University of Cambridge), is derived from PB-T-PAF [31] after the insertion of a woodchuck hepatitis virus (WHV) posttranscriptional regulatory element (WPRE) at the 3′ end. The pF5A_PBase plasmid encoding the piggybac transposase (PBase) was also generated by Dr Stephen Graham by subcloning the PBase sequence from pPBase [31] into the pF5A vector from Promega. The PB-RN plasmid carrying the reverse tetracycline transactivator (rtTA) inducer and the neomycin resistance gene was previously described [31]. The PB-RN, pF5A_PBase and one of the three PB-TRab27a plasmids were co-transfected into the Hela cells (ratio 1:1:5) using Lipofectamine 2000 (cat # 11668019; Thermo Scientific). After 16 h post-transfection, cells were treated with Puromycin (cat # P8833; Sigma-Aldrich) and Geneticin (cat. Numb. 11,811–031; Gibco) for one to two weeks to select cells. Different concentrations of Doxycycline (D9891; Sigma-Aldrich) were tested to induce Rab27a (WT or mutants) expression in those cells.

### Flow cytometry assays

Cell viability was assessed using flow cytometry with BD Horizon Fixable Viability Stain 575V (FVS575V) (BD Horizon, EUA, cat. 565694). The stock solution [200 μg of FVS575V in 340 μL of fresh cell culture-grade dimethyl sulfoxide (DMSO) (Sigma-Aldrich, San Luis, MI, USA)] was used to prepare the FVS575V working solution [1 μL of the stock solution in 1 mL of PBS]. HeLa cells were washed twice with ice-cold PBS, incubated with 100 μL of ice-cold FVS575V working solution for 15 minutes, and fixed with 100 μL of ice-cold 2% paraformaldehyde for 10 minutes. The cells were subsequently analyzed on the FACSymphony A5 Cell Analyzer (BD, Franklin Lakes, NJ, USA) at the Regional Blood Center of Ribeirão Preto—Center for Cell-based Therapy. For the gate strategy, cells were initially sorted based on side scatter and forward scatter parameters to identify singlets, followed by selecting live cells (negative for FVS575V). Data analyses were performed using the FlowJo software (https://www.flowjo.com/). To assay for surface CD4, HeLa cells were transfected with pEGFP-N1 (Clontec) and pCMV-CD4 [32]. After 20 h, the cells were incubated at 4°C with anti-CD4-APC (allophycocyanin)-conjugated antibody (Thermo Fisher Scientific, MHCD0405, S3.5 clone), for 40 minutes, and fixed with 2% paraformaldehyde for 10 minutes. Non-transfected HeLa cells were incubated with anti-CD4-APC (allophycocyanin)-conjugated and used to control for non-specific antibody labeling. APC fluorescence intensity data was acquired in cells expressing GFP using a FACSymphony A5 Cell Analyzer (BD, Franklin Lakes, NJ, USA). Data analysis was performed using the FloJo software (https://www.flowjo.com/).

### Coimmunoprecipitation of infected/transfected cells

For co-IP of infected cells, HeLa cells grown in 10 cm plates were infected with OROV (MOI = 4) and 4 hours after were transfected with 15 μg of plasmids expressing GFP-Rab27a or GFP (as a control). Cells were harvested at 24 h.p.i, lysed and processed following the protocol of GFP-trap agarose (Chromotek, Product code: gta). Briefly, cells were resuspended in lysis buffer [10 mM Tris/HCl pH 7.5, 150 mM NaCl, 0.5 mM EDTA, 0.5% Nonidet P40, supplemented with protease inhibitor cocktail (P8340; Sigma-Aldrich)] and centrifuged at 14000g for 20 min at 4°C. Protein levels in the clarified cell lysates were measured using the Bio-Rad’s Bradford protein assay (Biorad) to normalize total protein levels. Equal amounts of protein were incubated with GFP-Trap beads for 2 hours at 4°C under agitation followed by 5 washes with wash buffer (10 mM Tris/HCl pH 7.5, 150 mM NaCl, 0.5 mM EDTA), eluted with sample buffer [SDS 4%, Tris-HCl 160 mM (pH 6.8), glycerol 20%, DTT 100 mM and 0.005% Bromphenol Blue], boiled, and analyzed by SDS-PAGE and western-blot.

For co-IP of transfected cells, HEK293T cells grown in 10 cm plates were cotransfected with HA-Gn (7.5 ug) and HA-Gc (7.5 ug) and either GFP (15 ug) or GFP-Rab27a WT (15 ug), 24 hours after transfection cells were harvested, lysed and processed following the protocol of GFP-trap agarose (Chromotek, Product code: gta), as described above. Plasmids encoding OROV HA-tagged Gc and Gn were previously described [12].

## Results

### Characterization of OROV egress in HeLa cells

**Fig 1:**
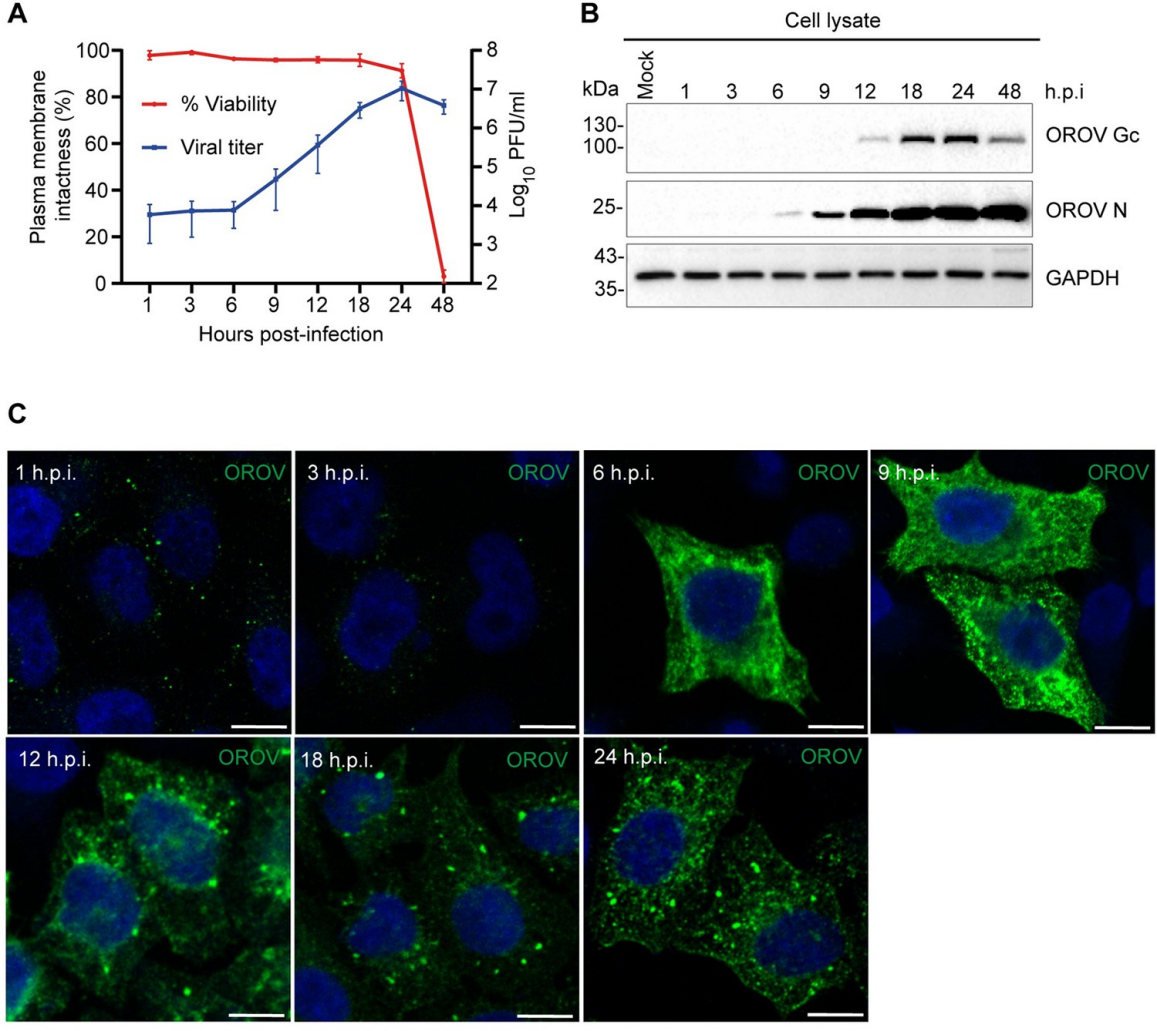
OROV replication cycle in HeLa cells. **(A)** Growth kinetics of OROV. HeLa cells monolayers were inoculated with OROV (MOI = 4) and the cell-free supernatant was collected at the indicated hours post-infection (h.p.i) and clarified. Infectious virus titers released into the cell culture media were quantified in Vero cells by plaque assay and are expressed as PFU/ml. In parallel, plasma membrane permeability in OROV-infected cells was measured by trypan blue exclusion from three independent experiments. Data represent the mean ± SD at indicated h.p.i. N = 3 **(B)** Intracellular viral proteins analyzed by western blot. HeLa cells monolayers infected as in (A) were processed for immunoblot at the indicated times post-infection. Detection of viral proteins was performed using mouse anti-OROV antiserum that recognizes Gc glycoprotein and N protein. Polyclonal rabbit anti-GAPDH antibody were used as a loading control of cell lysate samples **(C)** Monolayers of HeLa cells grown on coverslips was inoculated with OROV (MOI = 4) and fixed at the indicated post-infection times. The presence and intracellular distribution of the virus were monitored by indirect immunofluorescence, staining the cells with the anti-OROV same antiserum used for (B), followed by staining with a secondary anti-mouse IgG conjugated to Alexa Fluor 488 (in green). Nuclei are stained with DAPI (in blue) Scale bar: 10 μm.

We choose HeLa cells as a human cell-host model to study OROV particle release due to their susceptibility to OROV infection and excellent compatibility with transfection, RNAi, and high-resolution morphological analyses. To characterize the replication kinetics of OROV in this cell line, HeLa cells were infected at a multiplicity of infection (MOI) of 4 plaque-forming units (PFU), and samples of cell culture media were harvested at different times post infection, and the quantity of extracellular particles was determined by plaque assay (Fig 1A). The data indicate that OROV exit from cells is detectable approximately 9 hours post-infection (9 h.p.i.) and increases rapidly until 24 h p.i. A viability curve was also determined along the course of viral production. The result shows that up to 24 h.p.i., viral exit occurs in the absence of cell lysis, as the permeability of the cell’s plasma membrane was not significantly compromised when the virus accumulated extracellularly (Fig 1A).

In addition, the production of intracellular viral proteins over time was also determined by western blot, using an anti-OROV antiserum that detects both the Gc and the N proteins (Fig 1B). Concerning the production of intracellular viral proteins, the expression of Gc protein is detectable in cell lysates at 12 h.p.i. and after that, it increases until approximately 24 h.p.i., after which the intracellular levels seem to reduce slightly (S1A Fig). OROV N protein starts to be detected earlier in cell lysates, after 6 h.p.i., and also reaches plateau levels at approximately 18–24 h.p.i, but keeping stable levels in cells at least until 48 h.p.i (S1A Fig).

The subcellular distribution of viral proteins throughout the infection cycle was assessed by immunofluorescence, using the same anti-OROV antiserum (Figs 1C and S1B). At 1 h.p.i., viral protein labeling appears in isolated puncta scattered throughout the cytoplasm likely indicating endocytosed virions [10]. At 3 h.p.i. the number of OROV proteins positive puncta appeared to be lower, likely due to start of eclipse phase when the viral envelope fuses with the endosomal membrane to release viral genome segments into the cytoplasm (Figs 1C and S1B). At 6 h.p.i., the punctuated staining is lost from almost all cells and, instead a strong OROV signal dispersed throughout the cytoplasm is displayed by some cells (Figs 1C and S1B). This pattern likely indicates newly-synthesized N protein in the cytosol and glycoproteins in the endoplasmic reticulum (ER), [9,12] (S1B Fig). Between 9 to 12 h.p.i., the labeling pattern changes dramatically showing bright vesicle-like structures (Figs 1C and S1B) that may represent virus-induced cellular compartments that function as scaffolds for the assembly of viruses, termed viral factories [33]. Then, at 18 h.p.i. these viral factories become more prominent as the infection progresses until 24 h.p.i, when staining at the cell periphery was also observed. Importantly, at 18 h.p.i., almost all cells are clearly infected and displayed viral induced compartments (S1B Fig). Taken together, these results indicate that the majority of OROV release from infected cells occurs between 12 and 24 h.p.i., making this time frame the most suitable for OROV transport and egress studies in HeLa cells, which is the focus of this work.

### Rab27 GTPases are crucial for efficient OROV release from infected cells

**Fig 2:**
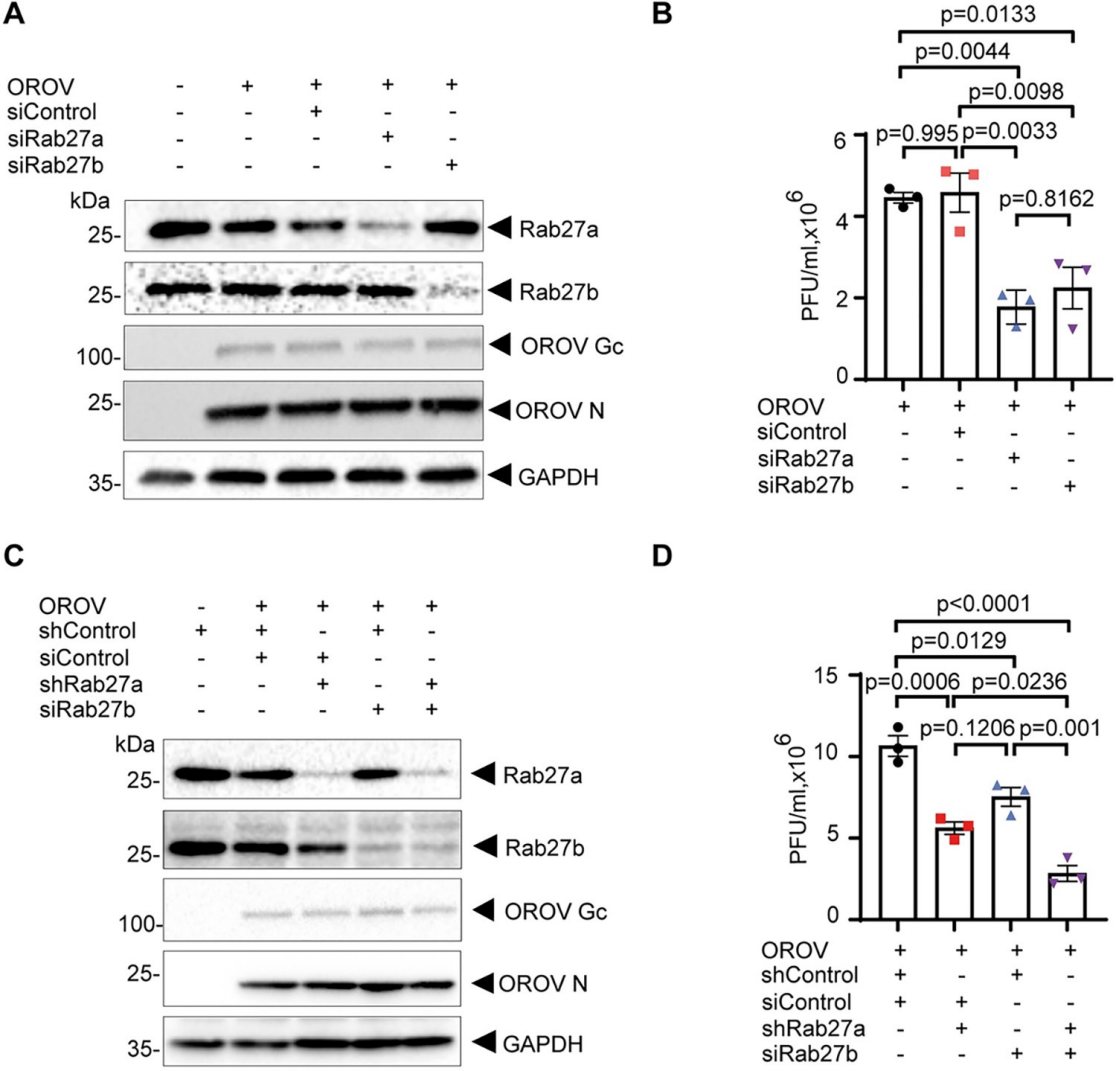
Rab27a knockdown impairs the release of OROV in HeLa cells. **(A)** HeLa cells were silenced for Rab27a or Rab27b using siRNA and later inoculated with OROV (MOI = 4). After 24 h, cells were lysed and subjected to western blot analysis using mouse anti-OROV antiserum, rabbit anti-Rab27a, rabbit anti-Rab27b and rabbit anti-GAPDH (used as a loading control). **(B)** HeLa cells conditioned media was used for viral titer determination by plaque assay for each condition. Data are the mean ± SD of three independent experiments. p>0.05 was considered as not significant (one-way ANOVA followed by Tukey´s multiple comparisons test). **(C)** HeLa shRab27a and shControl cells were mock transfected or transfected with control siRNA or Rab27b siRNA, as indicated, and later inoculated with OROV (MOI = 4). After 24 h, cells were lysed and subjected to western blot analysis using antibodies against indicated proteins. **(D)** The clarified supernatant of each condition was used for viral titer determination by the plaque assay method. Data are the mean ± SD of three independent experiments. p>0.05 was considered as not significant (one-way ANOVA followed by Tukey´s multiple comparisons test).

OROV assembly occurs at virally induced Golgi-derived compartments [12,13] that likely deliver newly synthesized particles to the cell periphery and mediate OROV cell egress. Since Rab27a and Rab27b GTPases were previously implicated in regulated secretion mediated by compartments of Golgi origin [20–22], we postulated that these GTPases contribute to OROV egress in a similar manner. To test this hypothesis, we knockdown (KD) the expression of either of the two Rab27 GTPases (Rab27a or Rab27b), which are encoded by different genes and share ~ 70% amino-acid identity [34]. Using specific siRNAs, a reduction of 85.96% (± 3.94%) and 85.16% (± 2.24%) in protein levels was achieved for Rab27a and Rab27b, respectively (Figs 2A and S2A). After siRNA treatment, cells were infected with OROV and examined at 24 h.p.i.. The levels of viral Gc and N proteins in cell extracts remained similar in either Rab27a or Rab27b knock down cells (Figs 2A and S2A), indicating that viral entry occurs when these GTPases are depleted.

In contrast, silencing of Rab27a or Rab27b significantly decreased infectious viral particles release in 59.81% (± 8.19%) and 52.51% (± 12.01%), respectively (Fig 2B). To confirm such finding, we generated a stable Rab27a KD HeLa cell line using shRNA (Fig 2C). Long term depletion of Rab27a in those cells did alter their viability in comparison to parental cells (S3A Fig) or the cell surface transport of transiently expressed CD4 (S3B Fig), indicating that constitutive secretory pathway is not significantly compromised by Rab27a depletion.

The shRNA-mediated depletion of Rab27a also resulted in a reduction of infectious OROV released into the media, showing a decrease of 47.31% (± 1.997%) (Fig 2D), without reducing the intracellular levels of viral proteins (Figs 2C and S2B). To test whether the detrimental effect on cell egress caused by Rab27a and Rab27b KD is additive, we use siRNA to KD Rab27b in the Rab27a KD cell line to simultaneously deplete both isoforms (Figs 2C and S2B). Although viral protein expression was unaffected, indicating that virus entry occurs, Rab27b and Rab27a co-depletion led to a 73.09% (± 8.82%) reduction in the release of infections OROV particles, an effect that was stronger compared to the individual depletion of the Rab27 isoforms (Fig 2D). Given that Rab27a or Rab27b depletion led to a reduction in OROV release and that we were unable to detect endogenous Rab27b for imaging analysis with the available antibodies, we decided to focus on Rab27a for the remainder of this study.

### Rab27a associates with OROV proteins and mediates transport the cell surface

**Fig 3:**
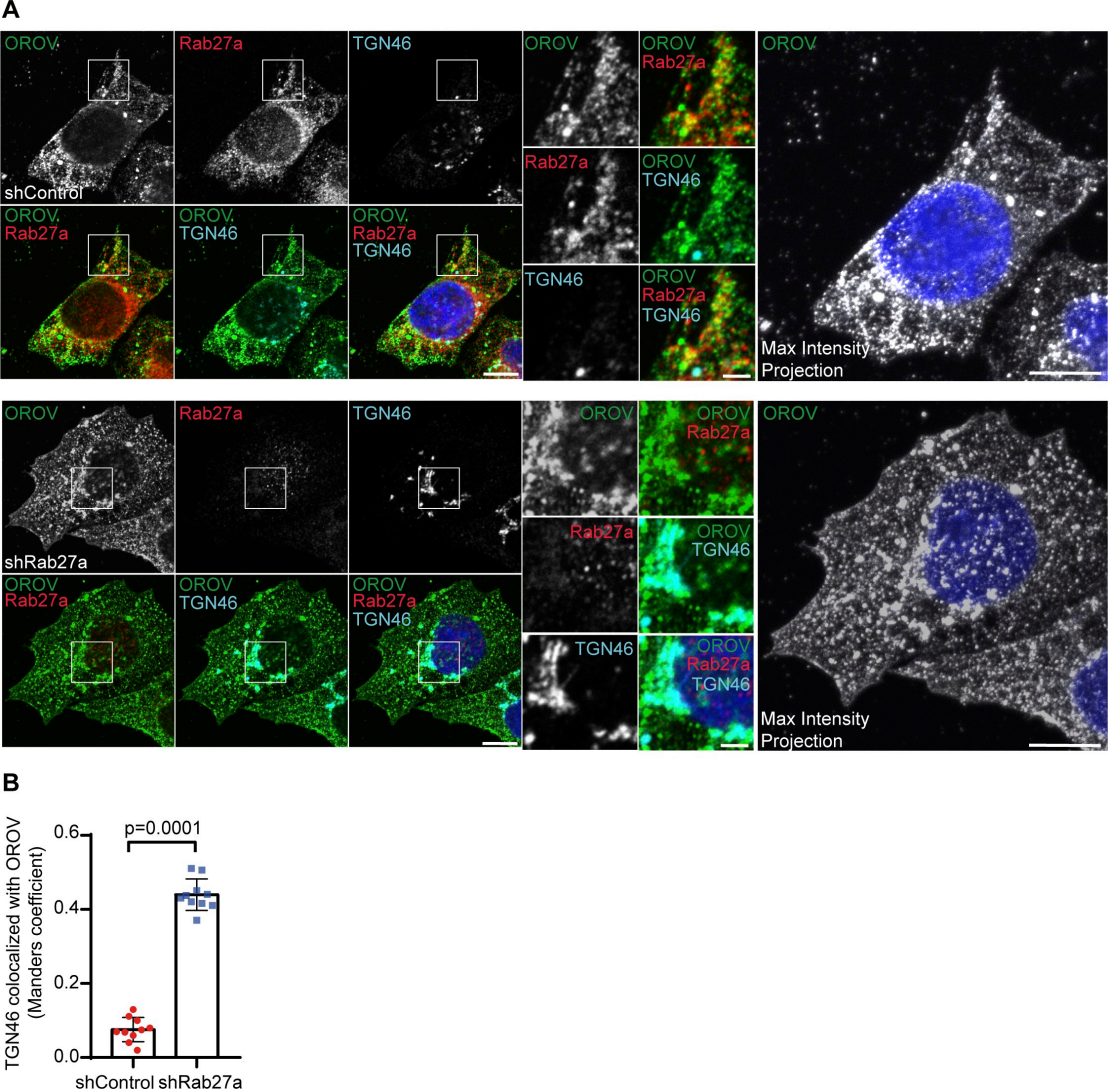
Rab27a colocalizes with OROV in late stages of infection. **(A)** HeLa shControl (upper panels) and shRab27a (lower panels) cells growing on coverslips were inoculated with OROV (MOI = 4) and fixed at 24 hours post-infection. Cells were permeabilized and stained with primary mouse anti-OROV, rabbit anti-Rab27a and sheep anti-TGN46 antibodies, followed by secondary anti-mouse IgG 488 (green), anti-rabbit IgG 594 (red) and anti-sheep IgG 647 (cyan). Nuclei are stained with DAPI (in blue). Scale bar = 10 μm. Insets represent the boxed areas. Scale bar = 2 μm. A maximum intensity projection image across the Z-stack for OROV staining is shown in each case. Scale bar = 10 μm. **(B)** Colocalization between TGN46 and OROV from the panel A, bars represent the mean ± SD of the Manders’ colocalization coefficient (at least 10 cells from three independent experiments). p>0.05 was considered as not significant (Unpaired t test).

Next, immunofluorescence assays were performed to investigate the colocalization of Rab27a with OROV proteins. As shown in S4A–S4B Fig, there is an increasing colocalization of Rab27a with structures containing OROV proteins as the infection progresses. Additionally, Rab27a is detected at the cell periphery of infected cells, in close proximity to OROV, at 24 hpi (S4A Fig, arrowheads). This result suggests the association of Rab27a to OROV structures mainly in the final stages of the replicative cycle. To get insights into the role of Rab27a in OROV externalization, we performed immunofluorescence assays of control or Rab27a KD HeLa cells infected with OROV (at 24 h.p.i.). In control cells, the colocalization of OROV with a TGN marker is low (Fig 3A and 3B), indicating that transport from the Golgi assembly sites to the plasma membrane for egress via the TGN is rapid and efficient, resulting in minimal accumulation of the virus in the TGN. In contrast, Rab27a depletion led to higher OROV colocalization with the TGN marker, suggesting that the loss of Rab27a function causes a buildup of viral material in the TGN (Fig 3A and 3B).

**Fig 4:**
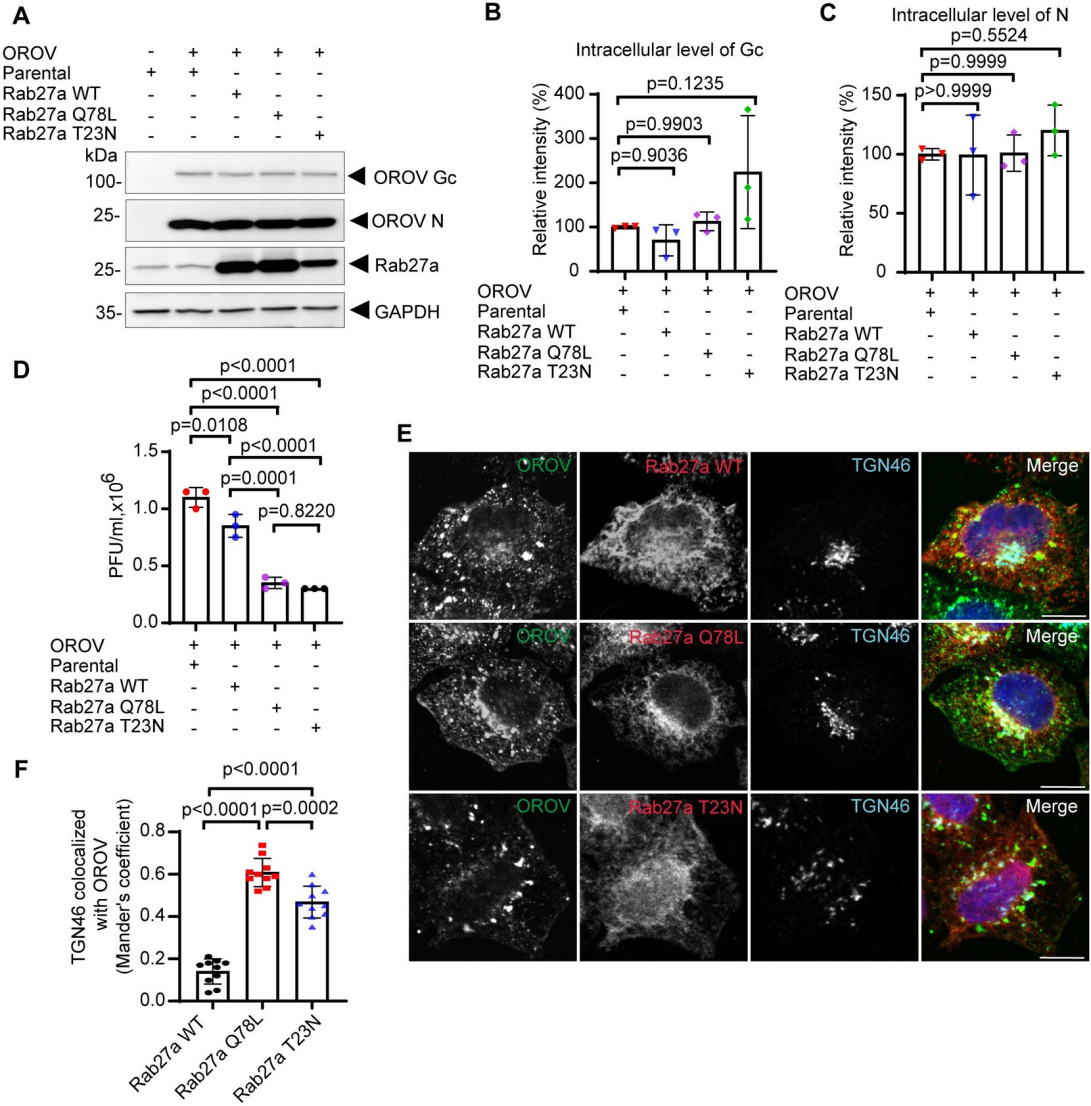
Overexpression of Rab27a mutants affects OROV intracellular distribution and release. **(A)** HeLa cell lines with inducible expression of Rab27a WT, Rab27a Q78L or Rab27a T23N mutants, or HeLa parental cells, were infected with OROV (MOI = 4) and after 4 hours the cells were treated with 10 ng/ml of Doxycycline to induce the expression. After 24 h.p.i., cells were lysed and subjected to western blot analysis using mouse anti-OROV (which recognizes N and Gc proteins), rabbit anti-Rab27a and rabbit anti-GAPDH used as a loading control. **(B-C)** Densitometry quantification of the Gc and N signals relative to GAPDH, as shown in panel A (n = 3 independent experiments). p>0.05 was considered as not significant (one-way ANOVA followed by Dunnett’s multiple comparisons test). **(D)** The clarified cell culture media of each condition in (A) were used for PFU assay. Data are the mean ± SD of three independent experiments. p>0.05 was considered as not significant. (one-way ANOVA followed by Tukey´s multiple comparisons test). **(E)** HeLa cell lines with inducible expression of Rab27a WT, Rab27a Q78L or Rab27a T23N mutants, growing on cover slips were infected with OROV (MOI = 4) and after 4 hours the cells were treated with 10ng/ml of Doxycycline to induce the expression. After 24 h.p.i., the cells were fixed, permeabilized and incubated with anti-OROV (mouse), anti-Rab27a (rabbit) and anti-TGN46 (sheep) antibodies, followed anti-mouse IgG 488 (green), anti-rabbit IgG 594 (red) and anti-sheep IgG 647 (cyan) secondary antibodies. Nuclei are stained with DAPI (in blue). Scale bar = 10 μm. **(F)** Colocalization between TGN46 and OROV from (E), bars represent the mean ± SD of the Manders’ colocalization coefficient (at least 10 cells from three independent experiments). p>0.05 was considered as not significant (one-way ANOVA followed by Tukey´s multiple comparisons test).

To further investigate whether OROV distribution is affected by Rab27 activity, we generated stable HeLa cell lines that overexpress exogenous Rab27a WT or either GTP-locked (Rab27a Q78L) or GDP-locked (Rab27a T23N) mutants, in a doxycycline inducible manner (Fig 4A). These GTP-locked and GDP-locked mutants, respectively act as dominant active and dominant inactive forms of Rab27a, disrupting Rab27a-mediated transport of vesicles and organelles when overexpressed [35–37]. The overexpression of Rab27a WT or the Rab27a mutants did not decrease the levels of intracellular OROV protein at 24 h.p.i (Fig 4A–4C). However, the levels of infectious particles released to the cell supernatants were reduced by 58.91% (± 1.06%) and 64.38% (± 2.44%) in cells overexpressing Rab27aQ78L or Rab27 T23N mutants, respectively (Fig 4D).

In addition, overexpression of either Rab27a mutant caused changes in the distribution of OROV staining compared to Rab27a WT, which displayed increased colocalization with a TGN marker (Fig 4E and 4F). Overall, the results suggest that Rab27a activity is required to efficiently transport nascent OROV particles from the Golgi area toward the cell surface for their release.

**Fig 5:**
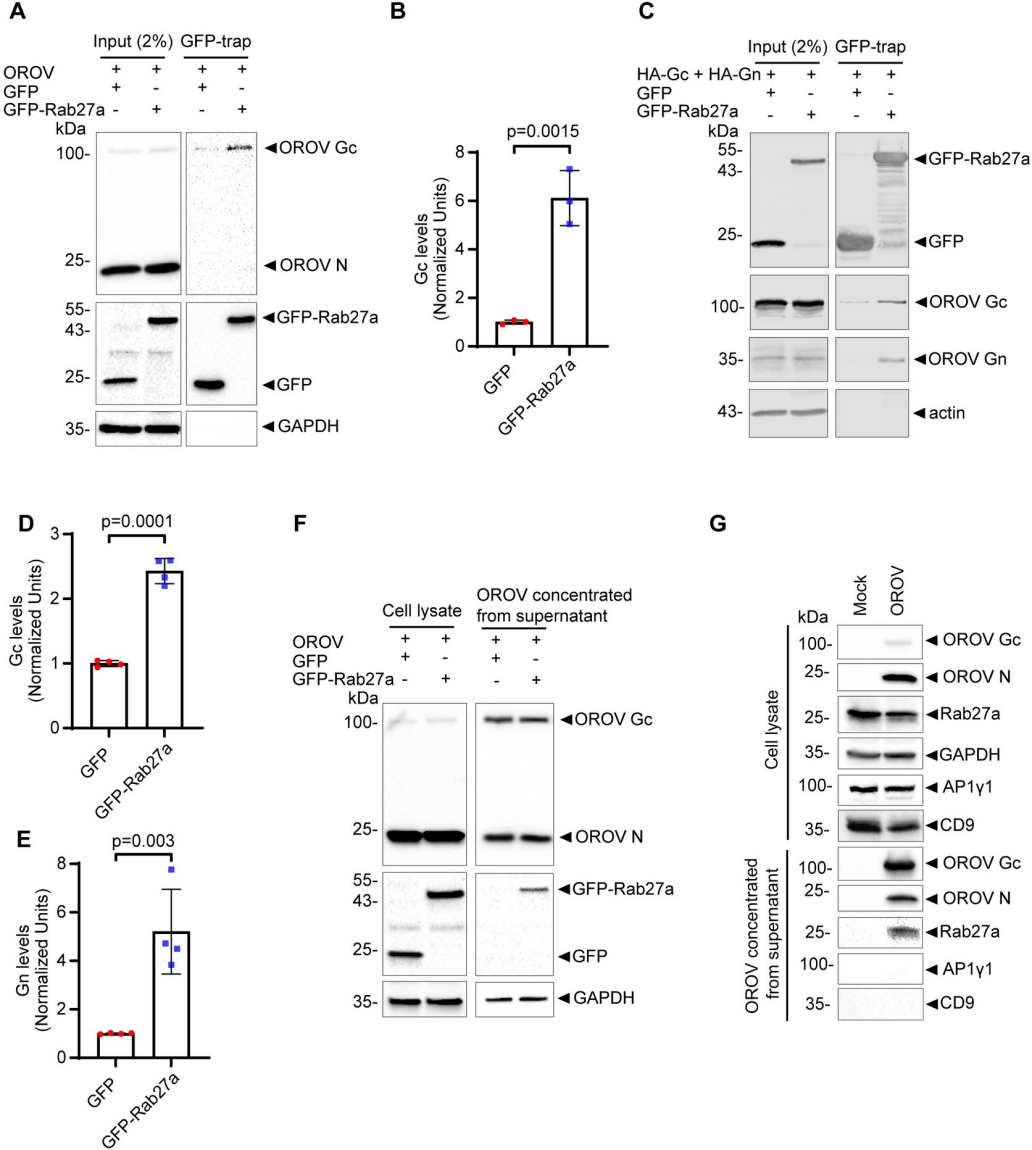
Rab27a interacts with OROV proteins and associates with the viral particles. **(A)** HeLa cells monolayers were infected with OROV (MOI = 4) and after 4 hours were transfected with plasmids expressing GFP-Rab27a and GFP as a control, 24 h.p.i. the cells were collected. The cells were lysed and subjected to affinity capture (IP) using GFP affinity matrices before western blot using mouse anti-OROV antiserum (that recognizes Gc and N proteins), rabbit anti-GFP and rabbit anti-GAPDH as a loading control. **(B)** Densitometry quantification of the amount of Gc immunoprecipitated by the GFP affinity matrices as shown in panel A (n = 3 independent experiments). p>0.05 was considered as not significant (Unpaired t test). **(C)** HEK293T cells monolayers were cotransfected with HA-Gn and HA-Gc plasmids and either GFP or GFP-Rab27a WT, 24 h after transfection, the cells were collected. The cells were lysed and subjected to affinity capture (IP) using GFP affinity matrices before western blot using rabbit anti-HA (to recognize Gc and Gn), mouse anti-GFP, and rabbit anti-actin as a loading control. **(D-E)** Densitometry quantification of the amount of Gc and Gn immunoprecipitated by the GFP affinity matrices as shown in panel C (n = 4 independent experiments). p>0.05 was considered as not significant (Unpaired t test). **(F)** HeLa cells monolayers were infected with OROV (MOI = 4) and after 4 hours were transfected with plasmids expressing GFP-Rab27a WT and GFP as a control, 24 hpi the cells and the supernatant were collected. The cells were lysed and subjected to western blot using the mouse anti-OROV, rabbit anti-GFP and rabbit anti-GAPDH as a loading control. The supernatant was ultracentrifuged (100000 x g for 90’ at 4°C) and subjected to western blot analysis using the same antibodies used for the cell lysate. **(G)** HeLa cells were infected with OROV (MOI = 4) or mock infected, 24 h.p.i. The cells and media were collected and virus particles were concentrated from the media by ultracentrifugation (100,000 × g for 90’ at 4°C). The lysates of cells and extracellular particles were analyzed by western blot using the antibodies indicated. AP1γ1 and CD9 were used as a cell cytoplasm contamination marker and as EVs marker respectively. p>0.05 was considered as not significant (Unpaired t test).

Next, we investigated whether Rab27a associates with viral components during replication. Initially, we demonstrated that OROV Gc is enriched in GFP-trap-based Co-IP assays using GFP-Rab27a as bait compared to GFP alone (Fig 5A and 5B). Interestingly, despite being highly present in the input, the N protein was not detectable in the GFP-Rab27a Co-IPs. To test whether the structural glycoproteins are sufficient viral factors for Rab27a interaction, we transiently expressed HA-tagged Gn and Gc (HA-Gn and HA-Gc) in HeLa cells in the absence of viral infection. Again, significantly higher levels of either HA-Gn and HA-Gc bound to GFP-Rab27a compared to GFP alone (Fig 5C–5E).

Additional evidence for OROV’s association to Rab27a is the fact that GFP-Rab27a, but not GFP, co-purified with OROV particles in ultracentrifugation pellets from supernatants of infected cells (Fig 5F), suggesting that Rab27a association with OROV proteins causes its co-release from cells. This notion was confirmed by the detection of endogenous Rab27a in OROV fractions obtained by ultracentrifugation of clarified supernatant of infected cells, contrasting with its absence in the ultracentrifugation pellet derived from the supernatant of mock-infected cells (Fig 5G). The Golgi/early endosome associated adaptor protein AP1γ1 or the extracellular vesicle (EV) marker CD9 were absent in those viral fractions, suggesting that the presence of Rab27a is not due to contamination with cytosolic material or increased general release of EVs caused by infection. Taken together, these data indicate the specific association of Rab27a to OROV components during particle biogenesis/egress.

### The Rab27a downstream effector Myosin Va is important for OROV trafficking and release

MyoVa is an actin-associated molecular motor known to interact with Rab27a [38]. Therefore, to confirm the involvement of Rab27a in OROV externalization and to gain insights into the underlying mechanism, the subcellular distribution of MyoVa was analyzed by immunofluorescence in the context of OROV infection. Similar to Rab27a, the immunofluorescence staining of MyoVa revealed a partial colocalization with OROV proteins, and this colocalization increased as the infection progressed (S5A and S5B Fig). Subsequently, we generated a MyoVa-depleted HeLa cell line using specific shRNA expression, achieving a reduction in MyoVa levels by 67.8% (± 6.64%) (S2C Fig). Importantly, we observed no significant alterations in cell viability or disruption in protein transport via the constitutive secretory pathway in these MyoVa KD cells compared to the parental cell line (S3 Fig).

**Fig 6:**
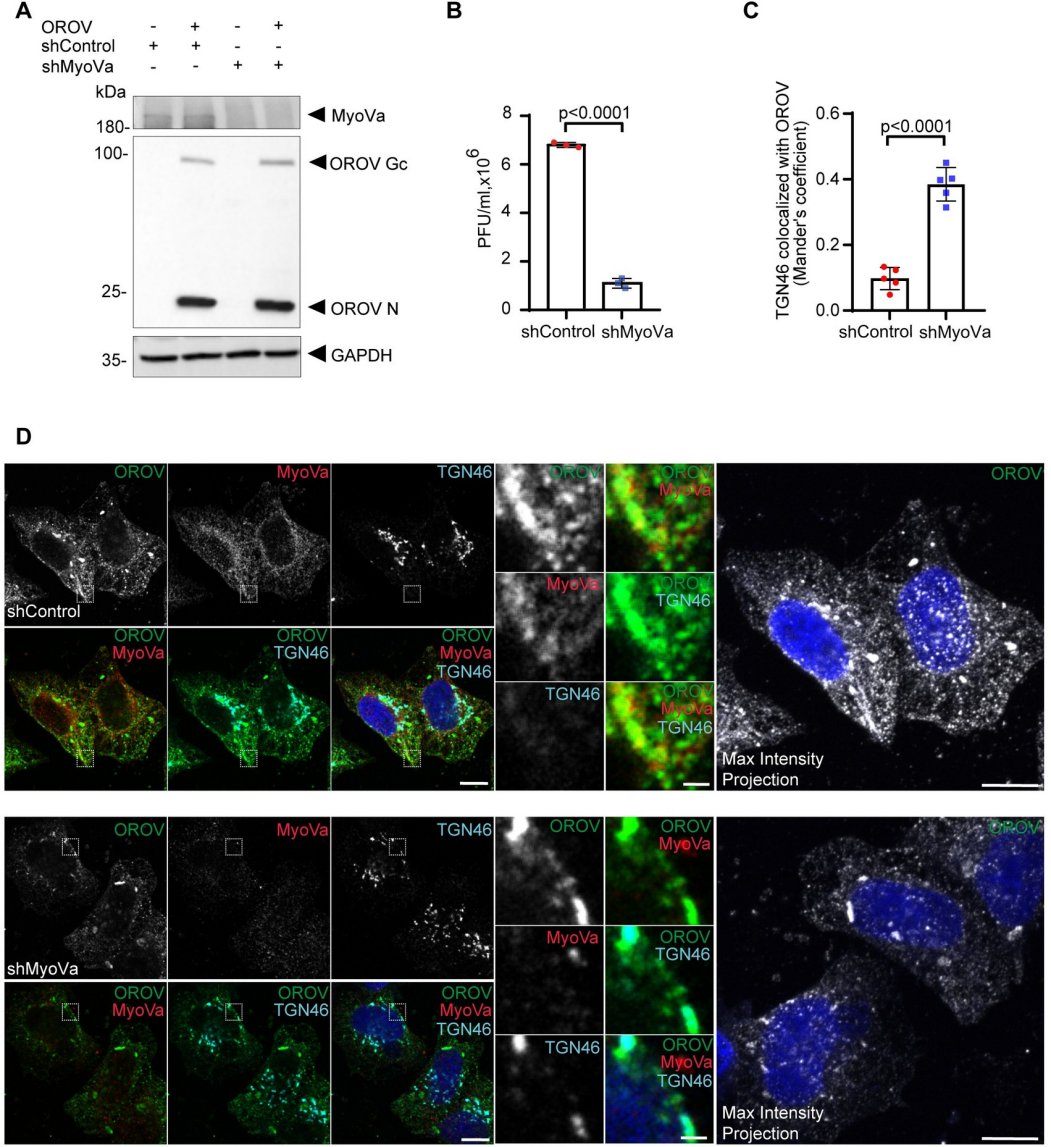
The Rab27a downstream effector Myosin Va is important for OROV trafficking and release. **(A)** HeLa shControl and shMyoVa cells were infected with OROV (MOI = 4) or mock infected. After 24 h, cells were lysed and subjected to western blot analysis using mouse anti-OROV, rabbit anti-MyoVa and rabbit anti-GAPDH used as a loading control. **(B)** The cell culture media of each condition was used for PFU assay. Data are the mean ± SD of three independent experiments. p>0.05 was considered as not significant (Unpaired t test). **(C)** Colocalization between TGN46 and OROV from (D), bars represent the mean ± SD of the Manders’ colocalization coefficient (at least 5 cells from three independent experiments). p>0.05 was considered as not significant (Unpaired t test). **(D)** HeLa shControl (upper panels) and shMyoVa (lower panels) cells growing on coverslips were inoculated with OROV (MOI = 4) and fixed at 24 h.p.i. Cells were permeabilized and stained with primary anti-OROV (mouse), anti-MyoVa (Rabbit) and anti-TGN46 (sheep) antibodies, followed by secondary anti-mouse IgG 488 (green), anti-rabbit IgG 594 (red) and anti-sheep IgG 647 (cyan). Nuclei are stained with DAPI (in blue). Scale bar = 10 μm. Insets represent the boxed areas. Scale bar = 2 μm. A maximum intensity projection image across the Z-stack for OROV staining is shown in each case. Scale bar = 10 μm. p>0.05 was considered as not significant (Unpaired t test).

Upon infecting the MyoVa KD cells, we did not detect notable alterations in the levels of intracellular viral proteins (Figs 6A and S2C). However, titration of infectious OROV in the supernatant from infected cells revealed a reduction of 83.85% (± 2.7%) in the amount of infectious OROV particles released from MyoVa KD cells compared to control cells (Fig 6B). This indicates that MyoVa plays a critical role in the efficient release of infectious OROV particles.

Immunofluorescence analysis of infected cells at 24 h.p.i. showed an increased colocalization of OROV with a TGN marker, suggesting a partial retention of virus particles near the TGN (Fig 6C and 6D). Additionally, there was a visible reduction in the presence of viral-induced compartments at the cell periphery in MyoVa KD cells compared to control cells (Fig 6D). Notably, the larger structures where viral proteins accumulate appeared more tubular in shape in MyoVa KD cells, which contrasts with the morphology observed in control cells (Fig 6D).

**Fig 7:**
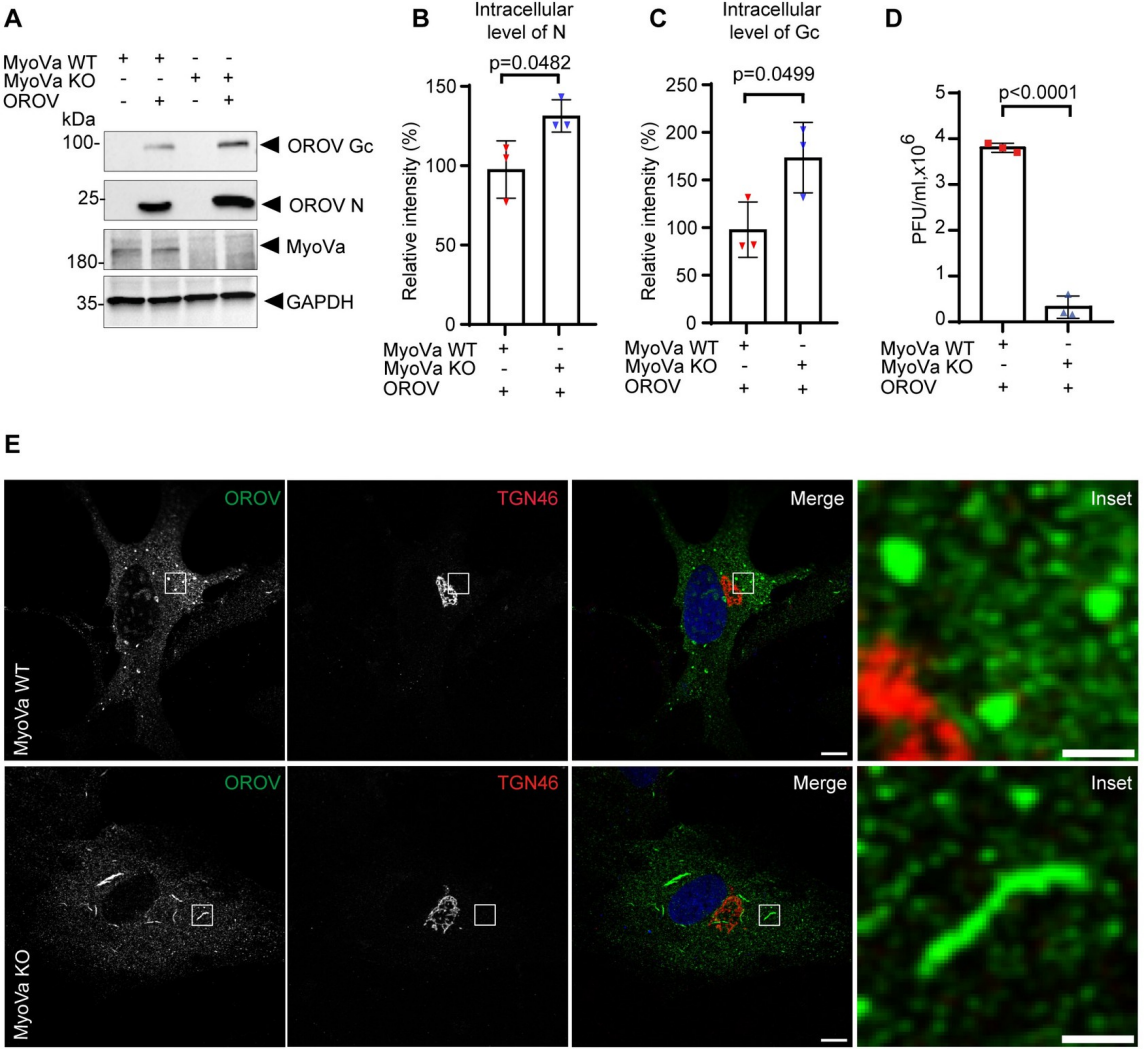
MyoVa KO Fibroblasts show alteration in the viral compartments’ morphology and a reduction in OROV release. **(A)** Control (MyoVa WT) and MyoVa-null (MyoVa KO) human fibroblasts were infected with OROV (MOI = 4) or mock infected. After 24 h, cells were lysed and subjected to western blot analysis using mouse anti-OROV (which recognizes N and Gc proteins), rabbit anti-MyoVa and rabbit anti-GAPDH used as a loading control. **(B-C)** Densitometry quantification of the amount of Gc and N as shown in panel A (n = 3 independent experiments). p>0.05 was considered as not significant (Unpaired t test). **(D)** The clarified cell culture media of each condition (panel A) were used for viral titer determination by plaque assay. Data are the mean ± SD of three independent experiments. p>0.05 was considered as not significant (Unpaired t test). **(E)** MyoVa WT and MyoVa KO fibroblasts growing on cover slips were infected with OROV (MOI = 4). After 24 h, the cells were fixed, permeabilized and incubated with anti-OROV (mouse) and anti-TGN46 (sheep) antibodies, followed anti-mouse IgG 488 (green) and anti-sheep IgG 594 (red) secondary antibodies. Nuclei are stained with DAPI (in blue). Scale bar = 10 μm. Insets represent the boxed areas. Scale bar = 2 μm.

The importance of functional MyoVa in OROV egress was further confirmed in MyoVa-null primary skin fibroblasts isolated from a patient with Griscelli Syndrome Type (Elejalde syndrome) [27]. Notably, infected MyoVa-null fibroblasts presented a small, but significant increase in the intracellular levels of viral proteins (Fig 7A–7C), but produced significantly lower amounts of infectious particles compared to normal skin fibroblasts (Fig 7D), a 91.5% (± 6.4%) reduction. Moreover, in comparison to control fibroblasts, the morphology of OROV compartments was clearly altered showing a tubular pattern (Fig 7E).

**Fig 8:**
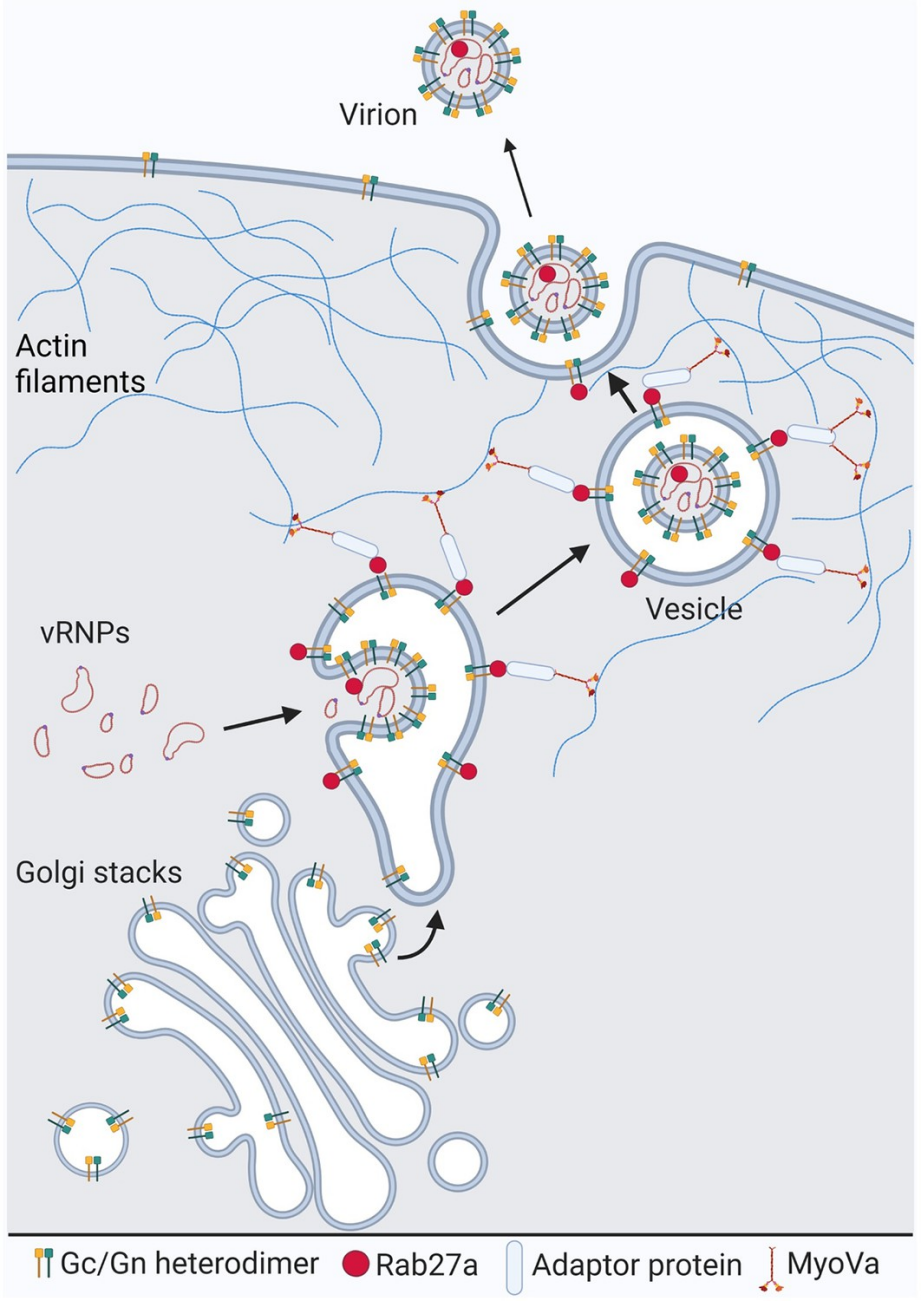
Proposed model for the Rab27a and Myosin Va mediated OROV cell egress. OROV envelope glycoprotein complexes are synthesized in the endoplasmic reticulum and transported to the Golgi, where virus assembly occurs for most orthobunyaviruses. The GTPase Rab27a may associate with the limiting membrane of these viral-induced Golgi/TGN-derived structures via interaction with OROV glycoproteins. This interaction likely occurs before the envelopment process is complete, occasionally resulting in the incorporation of Rab27a into nascent virions. The presence of Rab27a engages effector proteins, including Myosin Va, which propels the large virion-containing vesicles along actin filaments to the cell periphery. This movement continues across the actin cortical network, ultimately reaching the plasma membrane. At the plasma membrane, exocytic membrane fusion occurs, leading to the release of viral particles from the cells. Image created with BioRender.com.

Since MyoVa is a molecular motor that propels different cargos across actin filaments [39], we decided to test if these filaments are important for OROV transport to the cell periphery and egress. To this end, we used cytochalasin D (CYT), a drug that blocks actin filaments polymerization. OROV infected cells were treated with CYT at 14 h.p.i. and then fixed at 18 h.p.i.. This CYT treatment led to an accumulation of intracellular compartments containing viral proteins, and reduced viral staining at the cell surface. Interestingly, in CYT treated cells the viral induced compartments were surrounded by filamentous actin structures. The same phenotype was not observed in non-treated infected cells (S6A Fig). Finally, to investigate whether CYT treatment affected OROV cell release, the supernatant from both conditions was used to determine the viral titer (S6B Fig). Consistently, there was a significant reduction of 49.9% (± 2.5) in OROV release from CYT treated cells when compared to non-treated control cells. Taken together, our data suggest that Rab27a and its downstream effector Myosin Va are hijacked by OROV for efficient viral particles intracellular trafficking and release (Fig 8).

## Discussion

The mechanisms by which orthobunyaviruses undergo intracellular transport and egress from infected host cells are poorly characterized compared to viral entry and protein expression, as these early replication steps are more traditional targets of antiviral drugs and vaccines [40,41]. In addition, studying the last stages of the viral replicative cycle presents additional difficulties due to confounding impacts of cytopathic effects due to virus infection, especially in viruses that can induce apoptosis, such as OROV [42]. Although there are already reports showing virus growth curves for OROV in different cell lines [13,43], as we wanted to specifically study virus egress mechanisms it was important to monitor cell viability along the course of infection. This demonstrated that the majority of viral exit occurs between 12–24 h.p.i. in HeLa cells with only limited impacts on cell viability, providing a convenient time-frame to investigate viral release pathways (Fig 1).

Orthobunyavirus assembly is thought to start the Golgi complex [7,9,33]. Specifically, studies on OROV have shown that viral glycoproteins traffic from the ER to the Golgi, concentrating at Golgi membranes [12]. In this compartment, viral glycoproteins recruit ESCRT machinery components, which play a crucial role in viral production [12,13]. As viral particle assembly occurs, morphological changes in Golgi cisternae take place, resulting in the formation of vesicular compartments that are scattered throughout the cytosol and contain newly-formed OROV particles [13]. These Golgi-originated compartments are thought to mediate Orthobunyaviruses particles transport to the cell periphery and eventually fuse with the plasma membrane, allowing viral particles release [7,9]. However, the molecular mechanisms of Orthobunyaviruses egress still remain largely unknown.

Rab27 GTPases have been involved in secretion mediated by Golgi-originated secretory granules [20–22], lysosome-related organelles from various cell types [44–46], as well as, in the release of exosomes derived from intraluminal vesicles of multivesicular bodies [15]. The results in Fig 2 show that depletion of Rab27a and Rab27b individually, or combined, negatively affects infectious particle production. Under these ~85% knockdown conditions for each Rab27 isoform, we did not detect a consequent increase in the intracellular levels of Gc or N proteins (S2 Fig). One potential explanation for this apparent discrepancy is that only a small fraction of the viral proteins expressed in the cell are packaged into virions and subsequently secreted. Bunyaviruses are known to efficiently shut off host mechanisms, leading to high levels of viral protein expression within infected cells. We believe that most of the viral proteins produced do not get packaged into virions. However, the exact proportion of viral proteins that are secreted from bunyavirus-infected cells has not been determined, to the best of our knowledge. On the other hand, although our data does not completely rule out a delay in viral entry, the lack of Rab27 proteins do not seem to produce a detectable reduction in intracellular viral protein levels and formation of viral induced vesicles, suggesting that their activity is paramount for the late stages of OROV replicative cycle.

Moreover, Rab27 GTPases have already been reported to play important roles in the replication cycle of several viruses. Specifically, their activity is important for HSV-1 egress [23,24], HCV genome replication and expression [47], HCMV assembly [48], HEV release [25,26] and in HPIV-2 assembly [49]. Nevertheless, up to date, there are no published data reporting the involvement of the Rab27 activity in the replication cycle of bunyaviruses.

The immunofluorescence analysis shows evidence of increased colocalization between Rab27a and OROV proteins in an infection time dependent manner (S3 Fig). Interestingly, colocalization between OROV and Rab27a at the cell periphery is more prominent at the late stages of infection (24 h.p.i.). These results, combined with the data showing that inhibition of Rab27a function promotes colocalization of OROV with a TGN marker and reduces the release of infectious OROV into the culture media (Figs 3 and 4), suggest that OROV uses Rab27a-dependent trafficking pathways to reach the plasma membrane and exit the cell. In a potentially related pathway, Rab27a has been proposed to facilitate the docking and fusion of MVBs with the cell membrane for the extracellular release of exosomes [15,17].

The interaction we have identified between Rab27a and OROV glycoprotein (Fig 5) suggests a possible mechanism of recruitment of Rab27a to the viral compartment that contains newly-formed viral particles. This may be akin to other examples of viral proteins interacting with cellular proteins in order to facilitate their egress without cell lysis, as in the case of Kinesin-1 by the HSV-1 proteins pUS9 and pUS11 [50,51], the recruitment of kinesin-3 by PRV proteins pUS9 and the heterodimer gE-gI [52], the recruitment of MyoVa for HSV-1 [53] and the recruitment of Rab27a by HCMV [48]. Interestingly, we also observed the potential incorporation of Rab27a into OROV particles (Fig 5B). It is plausible to consider that this incorporation may be due to the localization of Rab27a to OROV assembly compartment due to interaction with the viral glycoproteins, leading to occasional packaging into virions during the virus budding step (Fig 8).

Importantly, in addition to a role for Rab27 in OROV egress, we have further demonstrated that the Rab27 effector MyoVa colocalizes with OROV positive structures over the course of infection (S5 Fig), and the inhibition of OROV release in MyoVa depleted cells (Figs 6 and 7), similar to the observed for HSV-1 egress [53]. Disruption of actin polymerization also alters the distribution of OROV positive compartments and inhibits virus release (S6 Fig), indicating that OROV requires active remodeling of actin filaments to exit cells, as suggested for the *Orthobunyavirus bunyamweraense* (BUNV) [54]. Interestingly, in MyoVa-null (MyoVa KO) human fibroblasts, in which the reduction of infectious OROV released reached more than 90%, a slight increase in intracellular levels of N and Gc was detectable (Fig 7).

To conclude, we proposed a model whereby Rab27a associates to the membrane of virus-induced vesicular compartments containing newly assembled OROV particles via interaction with the OROV glycoproteins. Rab27a association may take place in TGN-derived compartments, before the envelopment process is complete, which in turn recruit effectors proteins including MyoVa that propels the large virion-containing vesicles via actin filaments to the cell periphery and across the actin cortical network to reach the plasma membrane for exocytic membrane fusion and release from the cell (Fig 8).

## Supporting information

S1 Fig Western blots quantification and low magnification immunofluorescence imagens of the OROV replication curve displayed in Fig 1. **(A)** Densitometry analysis of the amount of Gc and N signals from western blots as shown in the Fig 1B (n = 3 independent experiments). **(B)** Monolayers of HeLa cells grown on coverslips were inoculated with OROV (MOI = 4) and fixed at the indicated times post-infection. The presence and intracellular distribution of the virus were monitored by indirect immunofluorescence, staining the cells with a mouse anti-OROV antiserum, followed by staining with a secondary anti-mouse IgG conjugated to Alexa Fluor 488 (in green). Nuclei are stained with DAPI (in blue) Scale bar: 10 μm.
(TIF)

S2 Fig Western blots quantifications of the Rab27a, Rab27b and Myosin Va Knockdown experiments in HeLa cells displayed in Figs 2 and 6. **(A)** Densitometry analysis of the amount of Rab27a, Rab27b, Gc and N signals as shown in Fig 2A (n = 3 independent experiments). p>0.05 was considered as not significant. One-way ANOVA followed by Tukey´s multiple comparisons test (for the case of Rab27a and Rab27b) and one-way ANOVA followed by Dunnett’s multiple comparisons test (for the case of Gc and N). **(B)** Densitometry quantification of the amount of Rab27a, Rab27b, Gc and N as shown in Fig 2C (n = 3 independent experiments). p>0.05 was considered as not significant. One-way ANOVA followed by Tukey´s multiple comparisons test (for the case of Rab27a and Rab27b) and one-way ANOVA followed by Dunnett’s multiple comparisons test (for the case of Gc and N). **(C)** Densitometry quantification of the amount of MyoVa, Gc and N as shown in Fig 6A (n = 3 independent experiments). p>0.05 was considered as not significant. One-way ANOVA followed by Tukey´s multiple comparisons test (for the case of MyoVa) and Unpaired t test (for the case of Gc and N).
(TIF)

S3 Fig Assessment of viability and cell surface protein levels in HeLa Rab27a and MyoVa knockdown (KD) cell lines via flow cytometry. Control HeLa cells (shControl) and HeLa cells knockdown for Rab27a (shRab27a) or Myosin Va (shMyoVa) were analyzed for cell viability and CD4 cell surface levels. **(A)** Dot plots show the population of viable HeLa cells (shControl, shRab27a or shMyoVa) by flow cytometry using the FVS575V reagent. **(B)** HeLa cells (shControl, shRab27a or shMyoVa) were transfected with pCMV-CD4 and pEGFP-N1. After 20 h, the surface levels of CD4 were analyzed by FACS. Histograms show the surface levels of CD4 in cells expressing GFP in each case. The histograms are representative of three independent experiments.
(TIF)

S4 Fig Analysis of the colocalization between Rab27a and OROV proteins along the infection. **(A)** HeLa cells grown on coverslips were inoculated with OROV (MOI = 4) and fixed at the indicated post-infection times. Cells were permeabilized and stained with primary mouse anti-OROV and rabbit anti-Rab27a antibodies, followed by secondary anti-mouse IgG 488 (in green) and anti-rabbit IgG 594 (in red). Nuclei are stained with DAPI (in blue). Scale bar = 10 μm. Insets represent the boxed areas. Scale bar = 2 μm. **(B)** Bars represent the mean ± SD of the Manders’ colocalization coefficient between OROV and Rab27a staining of at least 10 cells for each condition from three independent experiments. p>0.05 was considered as not significant (one-way ANOVA followed by Tukey’s multiple comparisons test).
(TIF)

S5 Fig Analysis of the colocalization between MyoVa and OROV proteins along the infection. **(A)** HeLa cells grown on coverslips were inoculated with OROV (MOI = 4) and fixed at the indicated times post-infection. Cells were permeabilized and stained with primary mouse anti-OROV and rabbit anti-MyoVa antibodies, followed by secondary anti-mouse IgG 488 (in green) and anti-rabbit IgG 594 (in red). Nuclei are stained with DAPI (in blue). Scale bar = 10 μm. Insets represent the boxed areas. Scale bar = 2 μm. **(B)** Bars represent the mean ± SD of the Manders’ colocalization coefficient between OROV and MyoVa staining of at least 10 cells for each condition from three independent experiments. p>0.05 was considered as not significant (one-way ANOVA followed by Tukey’s multiple comparisons test).
(TIF)

S6 Fig Disruption of actin filaments alters intracellular OROV distribution and egress from cells. **(A)** HeLa cells grown on coverslips were inoculated with OROV (MOI = 4) and at 14 h.p.i. cells were treated with either 4μM Cytochalasin D (CYT) or with DMSO as a control for 4h. At 18 h.p.i. the cells were fixed, permeabilized and stained with mouse anti-OROV antibody, followed by secondary anti-mouse IgG Alexa 488 (in green) and phalloidin 555 (in red). Nuclei are stained with DAPI (in blue). Scale bar = 10 μm. Insets represent the boxed areas. Scale bar = 2 μm. **(B)** The clarified supernatant was used for viral titer determination by the plaque assay. Data are the mean ± SD of three independent experiments. p>0.05 was considered as not significant (Unpaired t test).
(TIF)

S1 Data Excel Spread Sheet containing the numeric data for Figs 1A, 2B, 2D, 3B, 4B, 4C, 4D, 4F, 5B, 5D, 5E, 6B, 6C, 7B, 7C, 7D, S1A, S2A, S2B, S2C, S4B, S5B and S6B. (XLSX)
