# Supplementary figures and images for: Rab27a GTPase and its effector Myosin Va are host factors required for efficient Oropouche virus cell egress
Source: PLoS Pathog. 2024 Aug 30;20(8):e1012504. doi: 10.1371/journal.ppat.1012504 (PMC11392402; doi:10.1371/journal.ppat.1012504)

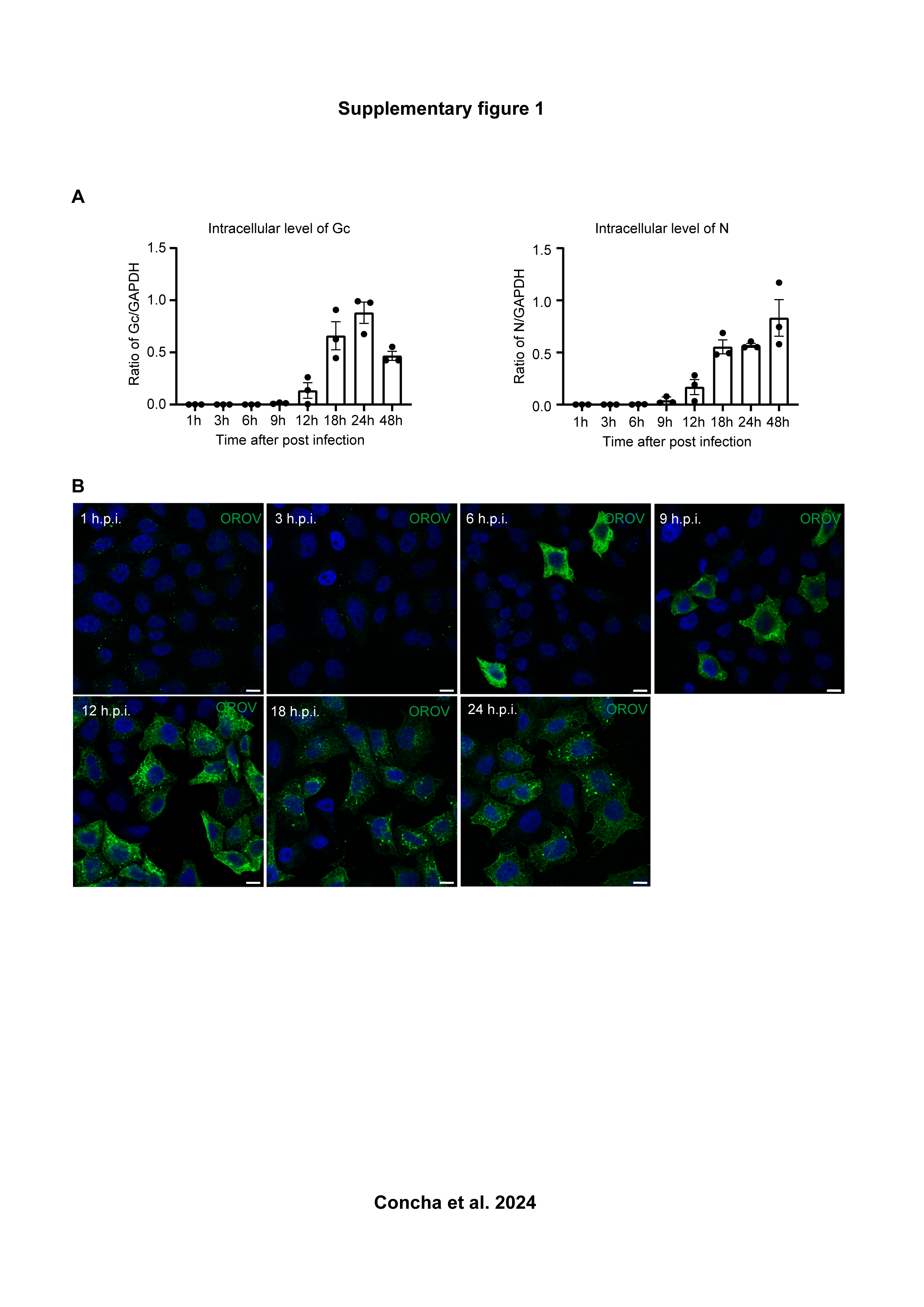

Supplement: S1 Fig — (A) Densitometry analysis of the amount of Gc and N signals from western blots as shown in the Fig 1B (n = 3 independent experiments). (B) Monolayers of HeLa cells grown on coverslips were inoculated with OROV (MOI = 4) and fixed at the indicated times post-infection. The presence and intracellular distribution of the virus were monitored by indirect immunofluorescence, staining the cells with a mouse anti-OROV antiserum, followed by staining with a secondary anti-mouse IgG conjugated to Alexa Fluor 488 (in green). Nuclei are stained with DAPI (in blue) Scale bar: 10 μm. (TIF) [file ppat.1012504.s001.tif]

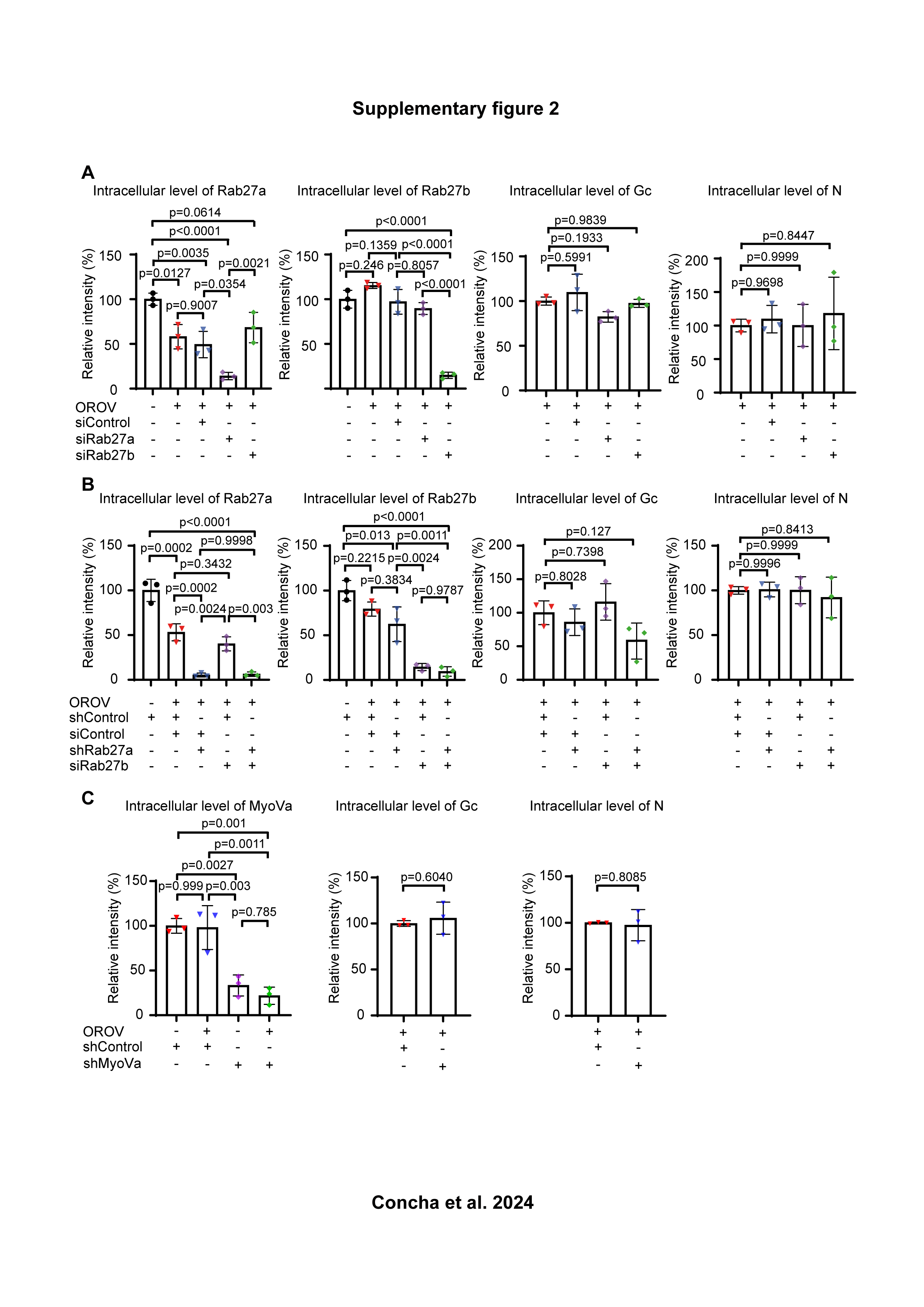

Supplement: S2 Fig — (A) Densitometry analysis of the amount of Rab27a, Rab27b, Gc and N signals as shown in Fig 2A (n = 3 independent experiments). p>0.05 was considered as not significant. One-way ANOVA followed by Tukey´s multiple comparisons test (for the case of Rab27a and Rab27b) and one-way ANOVA followed by Dunnett’s multiple comparisons test (for the case of Gc and N). (B) Densitometry quantification of the amount of Rab27a, Rab27b, Gc and N as shown in Fig 2C (n = 3 independent experiments). p>0.05 was considered as not significant. One-way ANOVA followed by Tukey´s multiple comparisons test (for the case of Rab27a and Rab27b) and one-way ANOVA followed by Dunnett’s multiple comparisons test (for the case of Gc and N). (C) Densitometry quantification of the amount of MyoVa, Gc and N as shown in Fig 6A (n = 3 independent experiments). p>0.05 was considered as not significant. One-way ANOVA followed by Tukey´s multiple comparisons test (for the case of MyoVa) and Unpaired t test (for the case of Gc and N). (TIF) [file ppat.1012504.s002.tif]

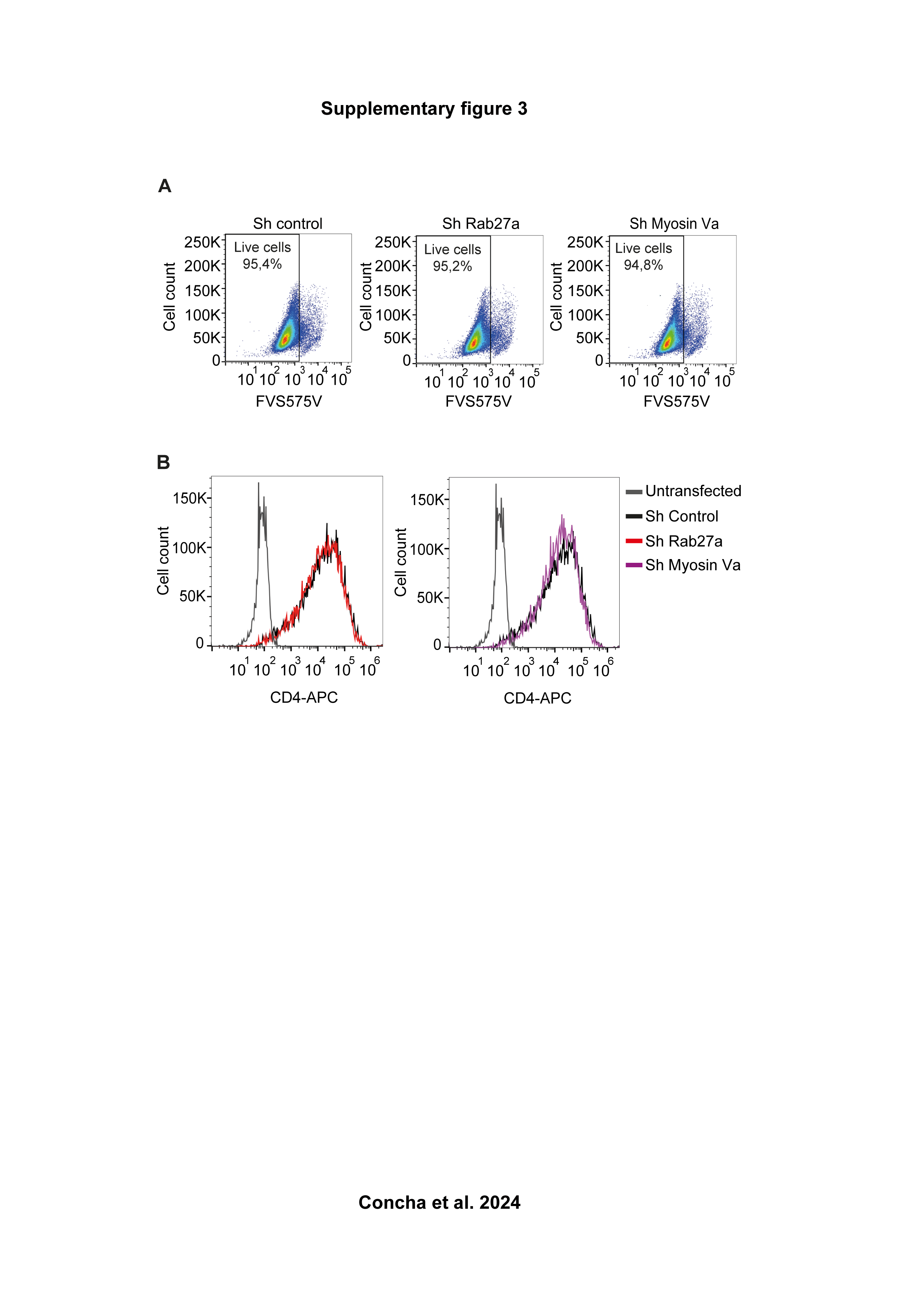

Supplement: S3 Fig — Control HeLa cells (shControl) and HeLa cells knockdown for Rab27a (shRab27a) or Myosin Va (shMyoVa) were analyzed for cell viability and CD4 cell surface levels. (A) Dot plots show the population of viable HeLa cells (shControl, shRab27a or shMyoVa) by flow cytometry using the FVS575V reagent. (B) HeLa cells (shControl, shRab27a or shMyoVa) were transfected with pCMV-CD4 and pEGFP-N1. After 20 h, the surface levels of CD4 were analyzed by FACS. Histograms show the surface levels of CD4 in cells expressing GFP in each case. The histograms are representative of three independent experiments. (TIF) [file ppat.1012504.s003.tif]

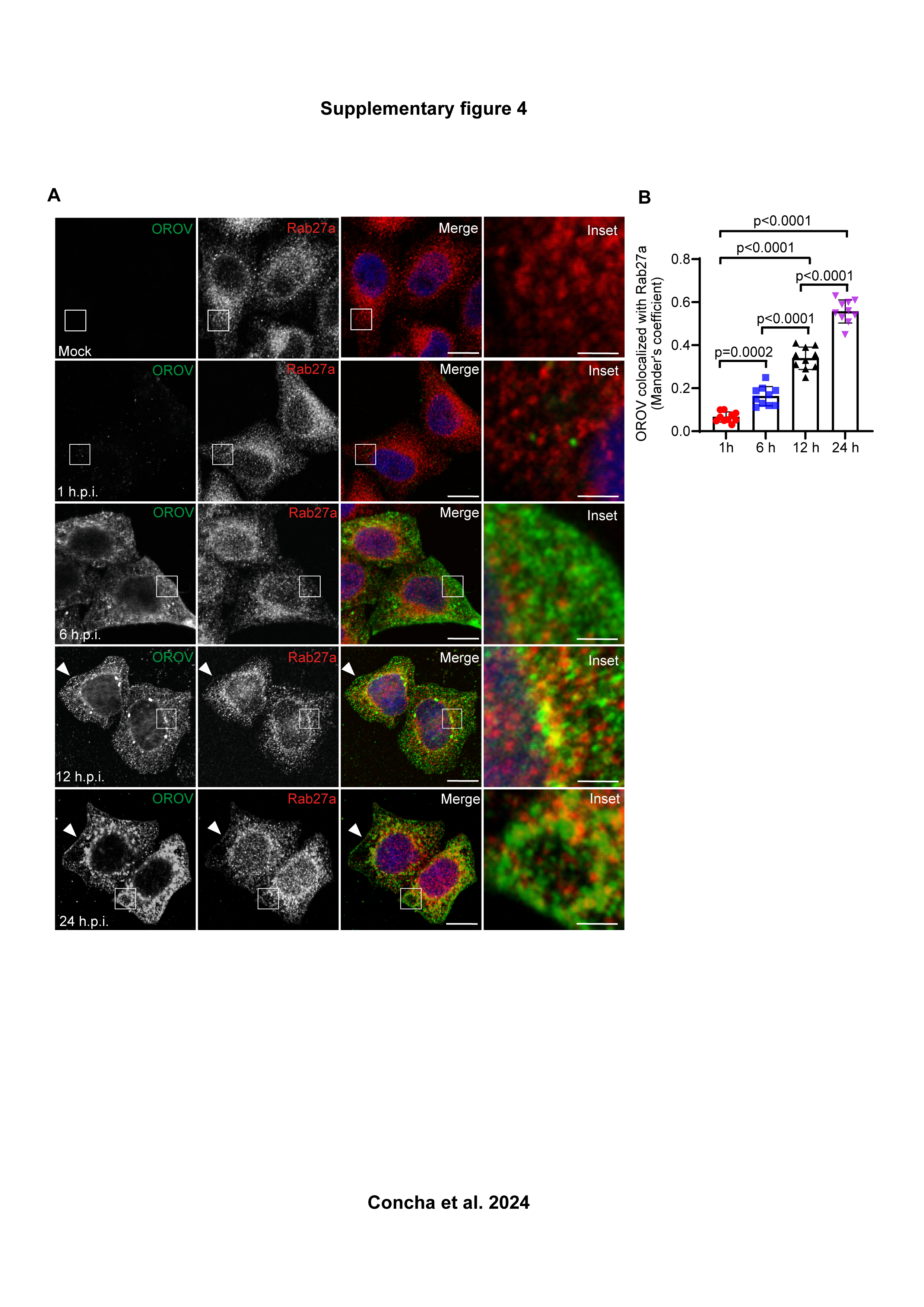

Supplement: S4 Fig — (A) HeLa cells grown on coverslips were inoculated with OROV (MOI = 4) and fixed at the indicated post-infection times. Cells were permeabilized and stained with primary mouse anti-OROV and rabbit anti-Rab27a antibodies, followed by secondary anti-mouse IgG 488 (in green) and anti-rabbit IgG 594 (in red). Nuclei are stained with DAPI (in blue). Scale bar = 10 μm. Insets represent the boxed areas. Scale bar = 2 μm. (B) Bars represent the mean ± SD of the Manders’ colocalization coefficient between OROV and Rab27a staining of at least 10 cells for each condition from three independent experiments. p>0.05 was considered as not significant (one-way ANOVA followed by Tukey’s multiple comparisons test). (TIF) [file ppat.1012504.s004.tif]

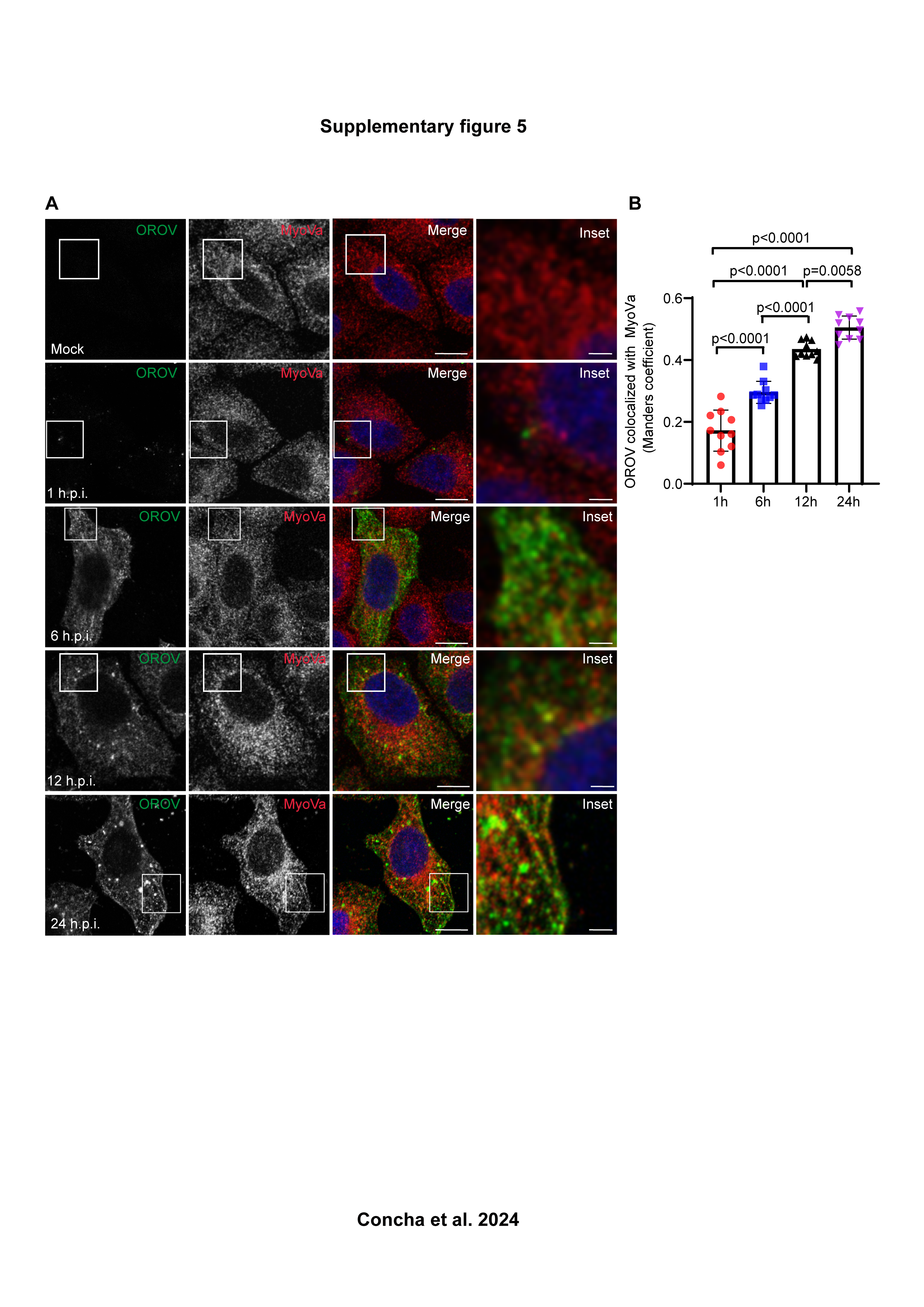

Supplement: S5 Fig — (A) HeLa cells grown on coverslips were inoculated with OROV (MOI = 4) and fixed at the indicated times post-infection. Cells were permeabilized and stained with primary mouse anti-OROV and rabbit anti-MyoVa antibodies, followed by secondary anti-mouse IgG 488 (in green) and anti-rabbit IgG 594 (in red). Nuclei are stained with DAPI (in blue). Scale bar = 10 μm. Insets represent the boxed areas. Scale bar = 2 μm. (B) Bars represent the mean ± SD of the Manders’ colocalization coefficient between OROV and MyoVa staining of at least 10 cells for each condition from three independent experiments. p>0.05 was considered as not significant (one-way ANOVA followed by Tukey’s multiple comparisons test). (TIF) [file ppat.1012504.s005.tif]

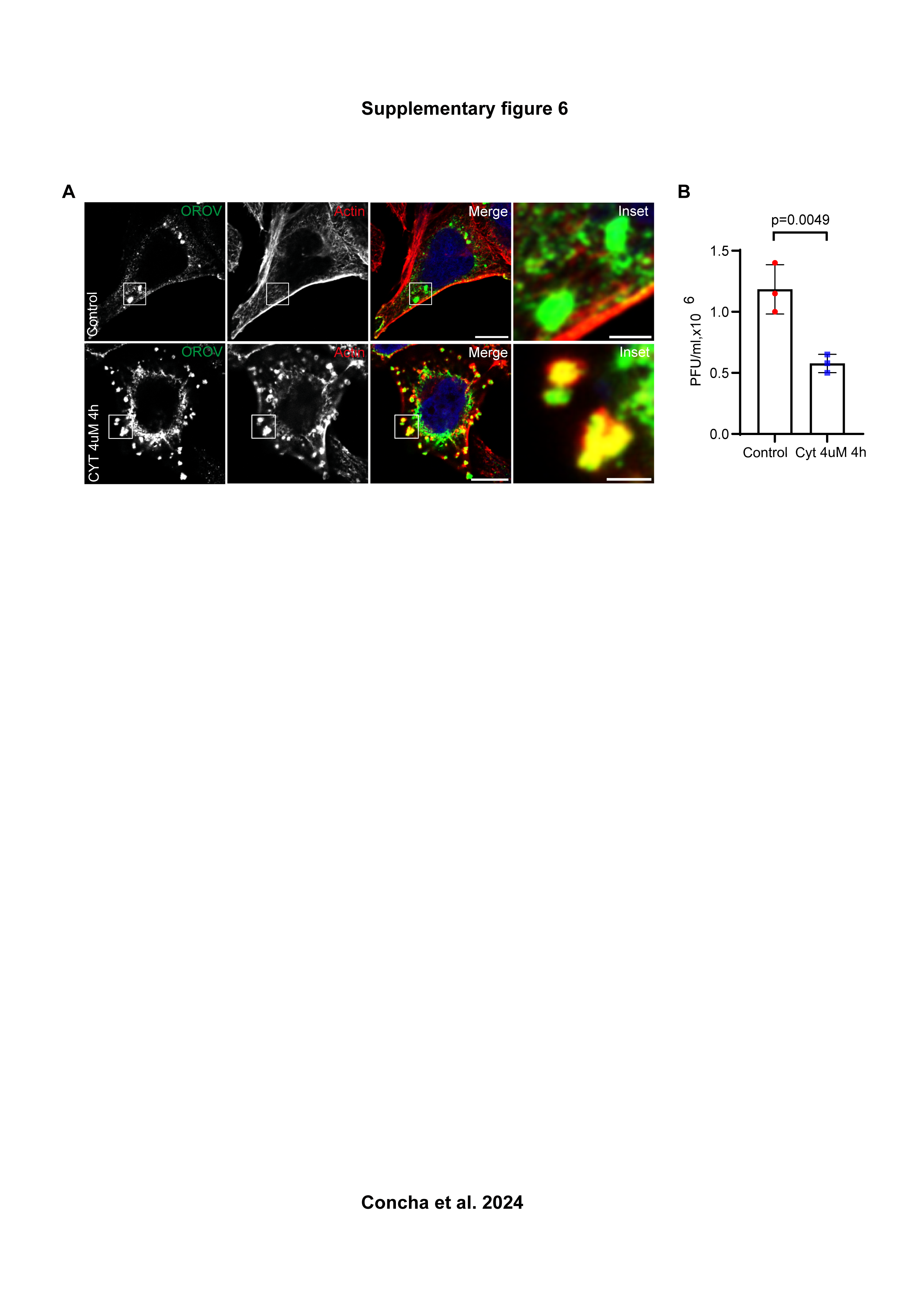

Supplement: S6 Fig — (A) HeLa cells grown on coverslips were inoculated with OROV (MOI = 4) and at 14 h.p.i. cells were treated with either 4μM Cytochalasin D (CYT) or with DMSO as a control for 4h. At 18 h.p.i. the cells were fixed, permeabilized and stained with mouse anti-OROV antibody, followed by secondary anti-mouse IgG Alexa 488 (in green) and phalloidin 555 (in red). Nuclei are stained with DAPI (in blue). Scale bar = 10 μm. Insets represent the boxed areas. Scale bar = 2 μm. (B) The clarified supernatant was used for viral titer determination by the plaque assay. Data are the mean ± SD of three independent experiments. p>0.05 was considered as not significant (Unpaired t test). (TIF) [file ppat.1012504.s006.tif]
